# NeuMapper: A scalable computational framework for multiscale exploration of the brain’s dynamical organization

**DOI:** 10.1162/netn_a_00229

**Published:** 2022-06-01

**Authors:** Caleb Geniesse, Samir Chowdhury, Manish Saggar

**Affiliations:** Biophysics Program, Stanford University, Stanford, CA, USA; Department of Psychiatry and Behavioral Sciences, Stanford University, Stanford, CA, USA

**Keywords:** TDA, Mapper, Optimal transport, Multitask fMRI, Ongoing cognition, NeuroSynth

## Abstract

For better translational outcomes, researchers and clinicians alike demand novel tools to distill complex neuroimaging data into simple yet behaviorally relevant representations at the single-participant level. Recently, the Mapper approach from topological data analysis (TDA) has been successfully applied on noninvasive human neuroimaging data to characterize the entire dynamical landscape of whole-brain configurations at the individual level without requiring any spatiotemporal averaging at the outset. Despite promising results, initial applications of Mapper to neuroimaging data were constrained by (1) the need for dimensionality reduction and (2) lack of a biologically grounded heuristic for efficiently exploring the vast parameter space. Here, we present a novel computational framework for Mapper—designed specifically for neuroimaging data—that removes limitations and reduces computational costs associated with dimensionality reduction and parameter exploration. We also introduce new meta-analytic approaches to better anchor Mapper-generated representations to neuroanatomy and behavior. Our new NeuMapper framework was developed and validated using multiple fMRI datasets where participants engaged in continuous multitask experiments that mimic “ongoing” cognition. Looking forward, we hope our framework will help researchers push the boundaries of psychiatric neuroimaging toward generating insights at the single-participant level across consortium-size datasets.

## Author Summary

Modern neuroimaging promises to transform how we understand human brain function, as well as how we diagnose and treat mental disorders. However, this promise hinges on the development of computational tools for distilling complex, high-dimensional neuroimaging data into simple representations that can be explored in research or clinical settings. The Mapper approach from topological data analysis (TDA) can be used to generate such representations. Here, we introduce several improvements to the underlying algorithm to aid scalability and parameter selection for high-dimensional neuroimaging data. We also provide new analytical tools for annotating and extracting neurobiological and behavioral insights from the generated representations. We hope this new framework will help facilitate translational applications of precision neuroimaging in clinical settings.

## INTRODUCTION

Modern noninvasive brain imaging technologies such as structural and functional magnetic resonance imaging promise to not only provide a better understanding of the neural basis of behavior but also to fundamentally transform how we diagnose and treat mental health disorders (Saggar & Uddin, 2019). However, unlike structural imaging, which has become standard in clinical practice, the clinical use of functional imaging (e.g., fMRI) has been limited to presurgical planning and functional mapping (Mitchell et al., 2013; Tie et al., 2014). One of the main reasons for the lack of fMRI-based clinical translation is that the traditional neuroimaging analyses (e.g., GLM or functional connectivity) tend to measure group-averaged (or central) tendencies, largely due to the low signal-to-noise ratio of the blood oxygenation level–dependent (BOLD) signal (Welvaert & Rosseel, 2013, 2014). Relatively newer functional connectome-based predictive modeling approaches have made some progress in generating insights at the single individual level (Bzdok et al., 2020; Shen et al., 2017), but several methodological issues need to be resolved before their clinical application becomes a reality (Dadi et al., 2019).

Recently, an approach called Mapper from the field of topological data analysis (TDA) has shown promise in generating data-driven insights from fMRI data at the single-participant level (Geniesse et al., 2019; Saggar et al., 2018). TDA is a recently developed field of mathematics that combines ideas from algebraic topology and network science (Carlsson, 2014), and TDA-based algorithms have gained recognition for their ability to generate robust, interpretable, and multiscale models of high-dimensional data (Giusti et al., 2016; Munch, 2017; Petri et al., 2014). Among these techniques, Mapper is a particularly successful method that produces a *shape graph*—a graphical representation of the underlying structure or shape of the high-dimensional data (Lum et al., 2013; Singh et al., 2007). Although Mapper bears some similarity to established dimensionality reduction methods (Belkin & Niyogi, 2003; Coifman & Lafon, 2006; Tenenbaum et al., 2000; Van Der Maaten & Hinton, 2008), it extends and improves upon such methods by (1) reincorporating high-dimensional information in the low-dimensional projection and thereby putatively reducing information loss due to projection, and (2) producing a compressed (and putatively robust) graphical representation of the underlying structure that can be analyzed using network science tools. The revealed graphical representation can also be annotated using meta-information to extract further insights about the underlying structure of the data. Analogous to how a geographical map encodes large-scale topographical features such as mountains, valleys, and plains, a shape graph produced by Mapper encodes essential topological features such as connectivity, adjacency, and enclosure. In the context of functional neuroimaging data, the shape graph encodes the higher order spatiotemporal features of brain activity that underlie cognition.

Mapper has been previously applied to generate insights from the underlying shape of data in oncology (Nicolau et al., 2011), transcriptomics (Rizvi et al., 2017), spinal cord and brain injury (Nielson et al., 2015), fragile X syndrome (Bruno et al., 2017; Romano et al., 2014), gene expression (Jeitziner et al., 2019), protein interaction (Sardiu et al., 2019), and materials science (Lee et al., 2017). In the field of neuroimaging, Mapper has been recently used to explore the whole-brain dynamics associated with different cognitive tasks and transitions during simulated “ongoing” cognition (Saggar et al., 2018); visualize the distributed and overlapping patterns of neural activity associated with different categories of visual stimuli via the DyNeuSR platform (Geniesse et al., 2019); and relate gene co-expression to brain function (Patania et al., 2019).

While initial neuroimaging applications of Mapper have been promising, several key methodological improvements to the processing pipeline are still needed, especially before the approach can be scaled up to larger consortium-style datasets. First, Mapper requires embedding the data into a low-dimensional space via a user-chosen target dimension *d* and filter function *f* : ℝ^*p*^ → ℝ^*d*^. Although the Mapper pipeline includes a partial clustering step to reincorporate some of the information loss due to initial projection (Singh et al., 2007), low-dimensional embedding is by definition an inefficient step due to an invariable loss of information by going down 2–3 orders of magnitude in dimensions. Second, the Mapper approach traditionally rescales the low-dimensional embedding to be inside a grid with overlapping cells. The size of the grid and the level of overlap are controlled by the resolution (*r*) and gain (*g*) parameters, respectively. A caveat with this construction is that the number of cells in a grid with fixed *r*, *g* grows exponentially in dimension *d*, leading to inefficient computations. Given recent evidence (and growing consensus) that large-scale consortium-level sample sizes are essential for accurately and reproducibly linking brain function and behavior (Marek et al., 2020), computational costs and scalability have thus become critical issues. Third, although Mapper results are stable over parameter perturbations, initial fine tuning of Mapper parameters is required due to their dependence on the data acquisition parameters (Saggar et al., 2018). Altogether, we argue that a systematic approach is required for exploring Mapper parameters, including *f*, *d*, *r*, and *g*, in order to select those that best capture the multiscale information putatively available in the neuroimaging data.

In this work, we provide significant methodological advances for each step of the Mapper processing pipeline and introduce novel approaches to generate neurobiological insights from the shape graphs. Hereinafter, we refer to our neuroimaging-focused Mapper pipeline as *NeuMapper*. Our framework moves away from dimensionality reduction altogether in favor of working directly with distance metrics in the original acquisition space, leading to a significantly faster pipeline that simultaneously avoids information loss due to low-dimensional projection. Toward optimizing parameter space exploration, we provide a semiautomatic parameter selection scheme using neuroimaging-specific objectives to remove all but a few parameter choices. Apart from the methodological advancements, we also introduce methods to generate novel neurobiological insights. For example, we introduce quantitative tools from computational optimal transport (OT) (Peyré & Cuturi, 2019) for better handling of overlapping graphical annotations as they take into account both global and local properties of the graph. Further, to better anchor the Mapper representations into cognitive neuroscience, we present a novel approach for annotating shape graph nodes using the NeuroSynth meta-analytic cognitive decoding framework (Yarkoni et al., 2011).

Using NeuMapper, we not only reproduce and independently validate the results obtained by the traditional Mapper approach (Saggar et al., 2018) but also reveal several new neurobehavioral insights. We show that individual differences in the mesoscale structure (e.g., modularity) of the NeuMapper-generated shape graphs could reveal important neurobehavioral insights. For example, in line with the previous work, we found that recruiting task-specific brain circuits led to better performance on the task. Further, applying tools from OT on shape graphs, we provide an avenue to study relations and dependencies between cognitive tasks. For example, we found that higher degree of overlap between brain circuits engaged during working memory and math is required for better performance on the math task. Lastly, by linking the NeuroSynth meta-analytic database with NeuMapper-generated shape graphs, we provide a new avenue to study and decode cognitively anchored changes in mental states at the highest temporal resolution. Here, we showed that such decoding could be helpful in revealing the negative impact of overreflection or attention lapses on task performance.

## RESULTS

Our results are grouped into three parts. In the first part (see section Methodological Advances in the NeuMapper Framework), we start by presenting the standard Mapper approach and the methodological advances in NeuMapper. In the next part (section Mesoscale Structure of Shape Graphs Informs Behavior), we show the relevance of mesoscale network statistics for deriving brain-behavior insights from shape graphs. In the final part (section Anchoring Shape Graphs Into Known Cognitive Constructs), we anchor shape graphs into cognitive topic terms using the meta-analysis framework of NeuroSynth (Yarkoni et al., 2011).

To test the efficacy of our NeuMapper approach, we used two independent fMRI datasets, of which the first was used for method development and the second was used as *held-out* data to be used only for final quantitative evaluation. In both datasets, participants performed a continuous multitask experiment with known ground truth about the timing of transitions between mental states as dictated by the task blocks. These datasets also contained task performance scores for each participant and hence could be used to ground Mapper-generated insights into behavior. Dataset 1 (*n* = 18) was previously collected by Gonzalez-Castillo et al. (2015) and comprised four task blocks (180 s each; repeated twice) consisting of resting state (R), working memory (M), math/arithmetic (A), and visual attention (V) tasks. We independently acquired Dataset 2 (*n* = 32) using the same paradigm as Dataset 1, but at a faster temporal resolution and shorter duration for task blocks (90 s; repeated twice). After discarding subjects due to excessive head motion and compliance issues resulting in very low behavioral scores, we retained 25 subjects in Dataset 2 (see Methods for details). We used Dataset 1 for methods development, leaving Dataset 2 aside for use only in the final quantitative analysis. Further, to demonstrate the robustness of our framework, we additionally performed reliability and validation checks via (1) extensive perturbation of Mapper parameters; and (2) comparison of results from real data with data generated from the phase-randomized null models.

### Methodological Advances in the NeuMapper Framework

We first begin by describing the traditional Mapper algorithm for generating a shape graph, followed by introducing two main improvements. Briefly, a shape graph is constructed from a dataset *X* ∈ ℝ^*p*^ via a four-step recipe: *filtering*, *binning*, *partial clustering*, and *graph generation*. In detail: (1) a dimension-reducing *filter* function *f* : ℝ^*p*^ → ℝ^*d*^ computes a low-dimensional embedding of *X*, (2) the embedding is covered by overlapping *d*-dimensional hypercubes; points in *X* are said to be in the same *cover bin* if their projections land in the same hypercube, (3) points in the same cover bin are further clustered into smaller *cluster bins* to account for faraway points (in *p*-dimensional space) erroneously landing in the same cover bin during projection, and (4) a graph is constructed with cluster bins as nodes, and edges between cluster bins that share points. Naively, the number of bins grows exponentially in dimension and becomes prohibitively expensive (Hinneburg & Keim, 1999; Sheikholeslami et al., 1998) to compute for *d* ≫ 2, thus putatively requiring conventional Mapper applications to rely on an initial embedding into no more than one or two dimensions. This poses a problem for neuroimaging data, where meaningful dimension reduction requires embedding dimensions ranging from five (Shine et al., 2019a) to 25 (Stevner et al., 2019) to 50 (Vidaurre et al., 2017). Additionally, exploring the large parameter space of Mapper is prohibitively expensive. While this issue could be mitigated by restricting the search space to fit within a preestablished computational budget, currently there are no neuroimaging-specific guidelines for obtaining this restricted search space.

**Figure 1.**
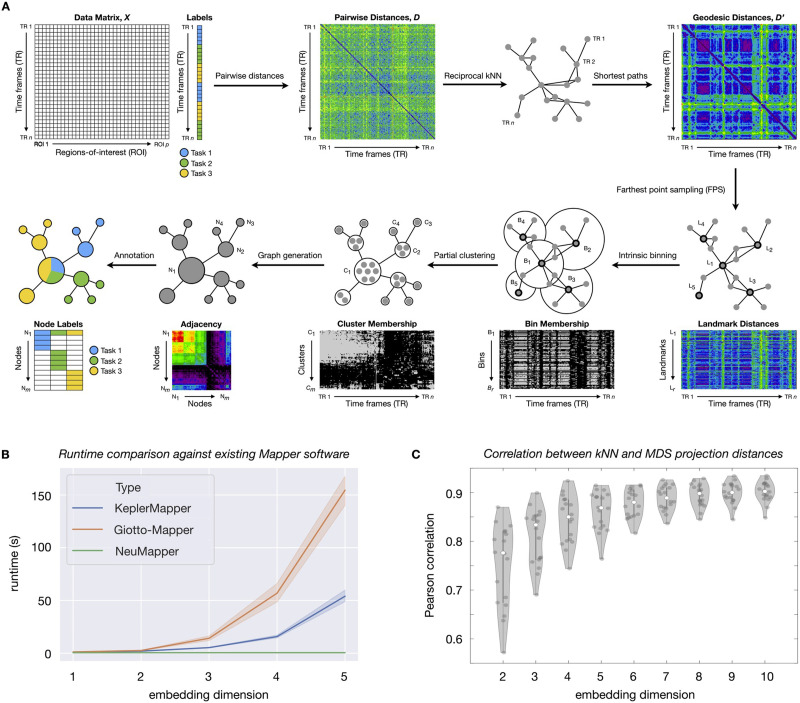
Methodological advances in the NeuMapper framework. (A) Given, a data matrix *X*, we first compute an initial distance matrix *D* and subsequently a reciprocal *k*NN graph. Next, we compute geodesic distances on this graph to obtain a transformed matrix *D*′ that captures nonlinear structure in the data. To access the hierarchical structure of this graph, we select landmarks *L*_1_, …, *L*_*r*_ on the data for a resolution parameter *r* and produce an overlapping partition with bins *B*_1_, …, *B*_*r*_ centered at these landmarks. Each bin is refined by a clustering step that uses the initial distances *D*, thus yielding a set of refined bins *C*_1_, …, *C*_*m*_ where *m* is possibly larger than *r*. Note that this step injects information from the native space of the data. Finally, a graph is constructed with nodes *N*_1_, …, *N*_*m*_ indexed by the refined bins and edges for overlapping bins. The nodes are further annotated by the labels of the data points, which may be user provided (e.g., different tasks in a blocked design, as shown here by Tasks 1–3) or generated by meta-analytic or otherwise data-driven approaches. (B) Open-source Mapper approaches (KeplerMapper 1.2.0 and Giotto-tda 0.2.2) explicitly construct a low-dimensional embedding and create hypercubes in the low-dimensional space. The complexity of this operation increases exponentially in dimension. NeuMapper avoids this step by performing implicit changes to the underlying distance matrix and can be much faster in practice. Here we standardized NeuMapper parameters to have the same y-intercept as KeplerMapper and Giotto-Mapper. Note also that KeplerMapper and Giotto-Mapper are written in Python and may obtain other benefits from interfacing with libraries such as scikit-learn. NeuMapper results reported here are from a version written in Matlab and meant to show only that the intrinsic binning method scales well with embedding dimension in practice. These results suggest that incorporating the NeuMapper methodology into KeplerMapper will allow the best of both pipelines. Differences in KeplerMapper and Giotto-Mapper runtimes are likely due to implementation differences, although we do not investigate these differences further. (C) Geodesic distances on the *k*NN graph can be embedded in Euclidean space using multidimensional scaling (MDS), but this projection step causes distortion. The amount of distortion goes down with increasing embedding dimension. However, using a high embedding dimension leads to costly computations in the standard Mapper approach. In contrast, our approach can work directly with the *k*NN distances and avoid this projection loss.

Our first methodological contribution (Figure 1A) is to modify the core Mapper algorithm to avoid relying on an explicit low-dimensional embedding. Instead, we start with a matrix *D* of distances (between whole or parcellated brain volumes) in the native high-dimensional space and produce a transformed matrix *D*′ that approximates the geometry of temporal trajectories through brain activity space. Specifically, we obtain *D*′ as geodesic distances on a *reciprocal* *k*-nearest neighbor (*k*NN) graph. This construction is reminiscent of a standard *k*NN graph, where data points are nodes and each point is connected to its *k*-closest neighbors, but the reciprocal variant adds an extra pruning step that reduces the effect of outliers (Qin et al., 2011). Supporting Information Figure S6 contains a toy example motivating our use of the reciprocal *k*NN. Next, we perform an *intrinsic* binning step that produces overlapping partitions of the data, but using only *D*′ and not any ambient space (for another application of this method, see Dłotko, 2019). Note that this intrinsic binning is a general way of scaling up computations and does not rely on a particular method of generating *D*′, so one could just as easily apply a moderate- to high-dimensional projection and calculate distances (e.g., Euclidean distances) to obtain *D*′. Our use of geodesic distances as above is not necessary for the framework, but effective for producing useful neurobiological insights. Overall, intrinsic binning simultaneously avoids high runtimes (Figure 1B) and projection-related information loss (Figure 1C) as compared to existing Mapper implementations that perform binning by constructing grids in the *d*-dimensional embedding space. Supporting Information Figure S1 provides a visual summary of the standard Mapper algorithm and adaptations for NeuMapper.

Our second methodological contribution is a semiautomated parameter selection framework that we developed to guide parameter exploration and selection. Specifically, we provide a heuristic algorithm that leverages the autocorrelation structure naturally present in fMRI data (due to the slow hemodynamic response) and returns a parameter choice that presents a mesoscale view—that is, between views that are “too local” or “too global”—of the data. See Methods for more details of our methodological contributions.

### Mesoscale Structure of Shape Graphs Informs Behavior

To ensure that our methodological advancements, for example, doing away with dimensionality reduction and utilizing an intrinsic binning strategy, can still recover previously reported neurobehavioral insights obtained using the traditional Mapper approach (Saggar et al., 2018), we first replicated initial results pertaining to the mesoscale properties of the shape graphs. Using our NeuMapper approach we not only replicated results based on the original dataset used by Saggar et al. (2018) (i.e., Dataset 1), but also reproduced the findings using an independent dataset (Dataset 2). In addition to replicating the mesoscale properties of the shape graphs, our NeuMapper approach can also capture temporal transitions in the brain activity patterns at the level of single time frames. In this section, we also show how our NeuMapper approach extends previous work using tools from OT theory to reveal pairwise mesoscale statistics.

Complex networks are often characterized by their hierarchical structure (Lancichinetti et al., 2009; Ravasz & Barabási, 2003), ranging from local descriptors at the node or edge level to global descriptors at the whole-graph level. At the mesoscale range are cohesive groups or clusters of nodes that are more densely connected to each other than to other nodes. In the most well-known model of these mesoscales, *community structure*, a group of nodes have higher density of within-group connections than a null model graph (Newman, 2006). A second, increasingly popular model is the *core-periphery structure*, where the network contains a dense core with high within-group connectivity that also occupy central positions in the network, and a periphery of nodes that are sparsely connected to each other (Borgatti & Everett, 2000; Rombach et al., 2014). Community and core-periphery structures have both been used extensively to gain insights into predictive components of functional brain networks, and new approaches into studying such mesoscale structures promise to deliver fundamentally new insights (Bassett et al., 2013; Sporns, 2013).

**Figure 2.**
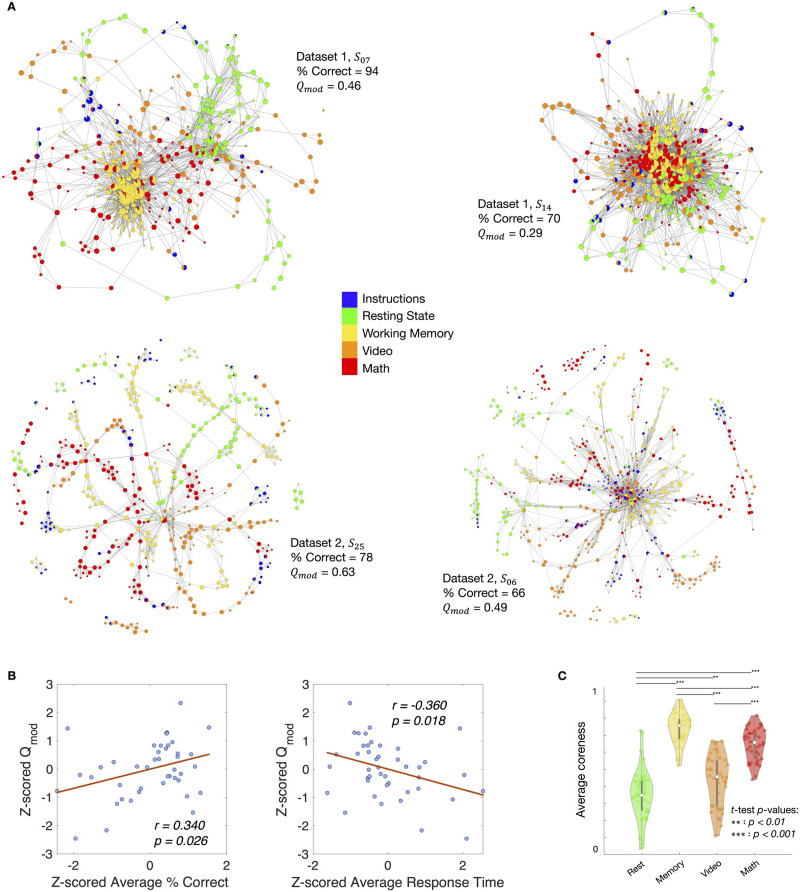
Mesoscale structure of shape graphs. (A) Shape graphs from two representative participants from Dataset 1 (*S*_07_ and *S*_14_) and two representative participants from Datset 2 (*S*_25_ and *S*_06_) colored by experimental task annotations. On the one hand, *S*_07_ from Dataset 1 is an example of a good performer (i.e., high average percent correct) and their shape graph had a highly modular structure (i.e., nodes are preferentially connected to other nodes associated with the same task). On the other hand, *S*_14_ from Dataset 1 is an example of a relatively worse performer (i.e., lower average percent correct) and their shape graph had a much less modular structure (i.e., nodes associated with different tasks are connected to each other without any preference for those associated with the same task). (B) Significant correlations between the modularity score (*Q*_*mod*_) and average task performance (e.g., percent correct, response time) were observed across both datasets (after *z*-scoring within each dataset, separately). These results replicate and strengthen the findings by Saggar et al. (2018) that higher modularity in the shape graph was associated with better task performance. (C) Separation between tasks as measured by core-periphery scores was observed across both datasets. These results further reproduce and strenghten the finding by Saggar et al. (2018) that nodes containing resting time frames mainly resided in the periphery (i.e., lower coreness scores), while nodes associated with the more cognitively demanding tasks (e.g., memory, math) tended to localize relatively deeper inside the shape graph (i.e., higher coreness scores).

Given a graph partition that yields communities, the modularity Q-score (*Q*_*mod*_) measures the quality of modularity or community structure (Newman, 2006). A higher *Q*_*mod*_ score implies better community structure. Previously, Saggar et al. (2018) annotated the shape graphs based on task blocks and computed the modularity using node-level task-based annotation as community assignment. Using Dataset 1, they found that participants whose shape graph had higher task-based modularity performed better across the tasks in the continuous multitask paradigm (CMP) (in terms of both accuracy and response time). This suggested that participants with task-specific functional activations performed better on average across different tasks. We first replicated this finding on Dataset 1 using our NeuMapper framework to ensure that our strategy of moving away from dimension reduction still recovers previously reported brain-behavior relations. Specifically, we observed a significant correlation between the average task performance (accuracy) and the task-based modularity of the shape graphs (*r* = 0.561, *p* = 0.015). Additionally, we observed a significant correlation between response time and modularity (*r* = −0.488, *p* = 0.040). We further validated the task accuracy (*r* = 0.340, *p* = 0.026) and response time (*r* = −0.360, *p* = 0.018) findings in a larger dataset, *n* = 43, combining participants from Datasets 1 and 2 (Figure 2B). Supporting Information Figure S2 shows the modularity-behavior correlations for the individual datasets. Further, to verify that these results cannot be reproduced via null models that preserve linear properties of the data, we carried out the analysis pipeline on phase-randomized null surrogates and observed that these correlations disappeared (Supporting Information Figure S3). Finally, to show that the modularity-behavior correlations described above are largely stable to parameter perturbation, we performed a grid search over a moderate region of parameter space surrounding the optimal parameter values for each dataset, and then computed modularity-behavior correlations across Datasets 1 and 2. We report heat maps of these correlations and their significance (*p* values) in Supporting Information Figure S4.

Next, we examined the core-periphery mesoscale structure of shape graphs. In the context of neural processes engaged during the CMP, core nodes could represent whole-brain configurations that consistently appear across a scan session, for example, due to task-switching in a CMP or due to high cognitive demands (Saggar et al., 2018; Shine et al., 2019a). To replicate previous finding that configurations from resting state are better represented by peripheral excursions, whereas those from high cognitive load are better represented by the core nodes, we estimated *coreness* scores for nodes in the shape graphs. We recover the core-periphery structure observed by Saggar et al. (2018), that is, one-way ANOVA revealed a significant effect of task in Dataset 1 (*F*(3, 68) = 67.0, *p* = 2.9 · 10^−20^) such that tasks with high cognitive load such as working memory or math were associated with nodes found relatively deep inside the core of the shape graphs, whereas resting-state nodes were relatively more peripheral. We additionally carried out the nonparametric Kruskal–Wallis test and observed a significant effect of task (*H*(3) = 55.8, *p* = 4.8 · 10^−12^). We further validated these findings on Dataset 2 using both one-way ANOVA (*F*(3, 96) = 35.6, *p* = 1.5 · 10^−15^) and the Kruskal–Wallis test (*H*(3) = 54.2, *p* = 1.0 · 10^−11^) with the same observation regarding working memory nodes being in the core and resting-state nodes being in the periphery. The results obtained by combining participants from Datasets 1 and 2 are shown in Figure 2C. Supporting Information Figure S2 shows the core-periphery structure for the individual datasets. Additionally, we observed that this core-periphery structure disappeared in the phase-randomized null surrogates (Supporting Information Figure S3).

As a final replication step, we also examined whether our NeuMapper approach can reveal transitions in task-evoked brain activity at the level of individual time frames. Given a shape graph, we follow Saggar et al. (2018) in constructing a (#time frames × #time frames) temporal connectivity matrix (TCM) that shows how each time frame is connected (or similar) to all other time frames in the graph. Using the traditional Mapper approach on Dataset 1, Saggar et al. (2018) found that time frames associated with tasks (e.g., working memory, video, math) typically had a higher degree of connectivity in the TCM, while those occurring between task blocks or during rest typically displayed a lower degree of connectivity. Further, they found that the temporal evolution of the degree connectivity (i.e., of each time frame, across the entire scan) recovered the task block structure (i.e., higher degrees evoked by and maintained during non-rest task blocks) and between-task transitions (i.e., lower degrees spanning the between-task instructional periods) of the CMP. We reproduced these previous findings on the same Dataset 1 using NeuMapper (Supporting Information Figure S5). Further validating our new framework, these results suggest that shape graphs produced by NeuMapper can capture similar temporal properties of the data compared to those produced by the traditional Mapper approach.

**Figure 3.**
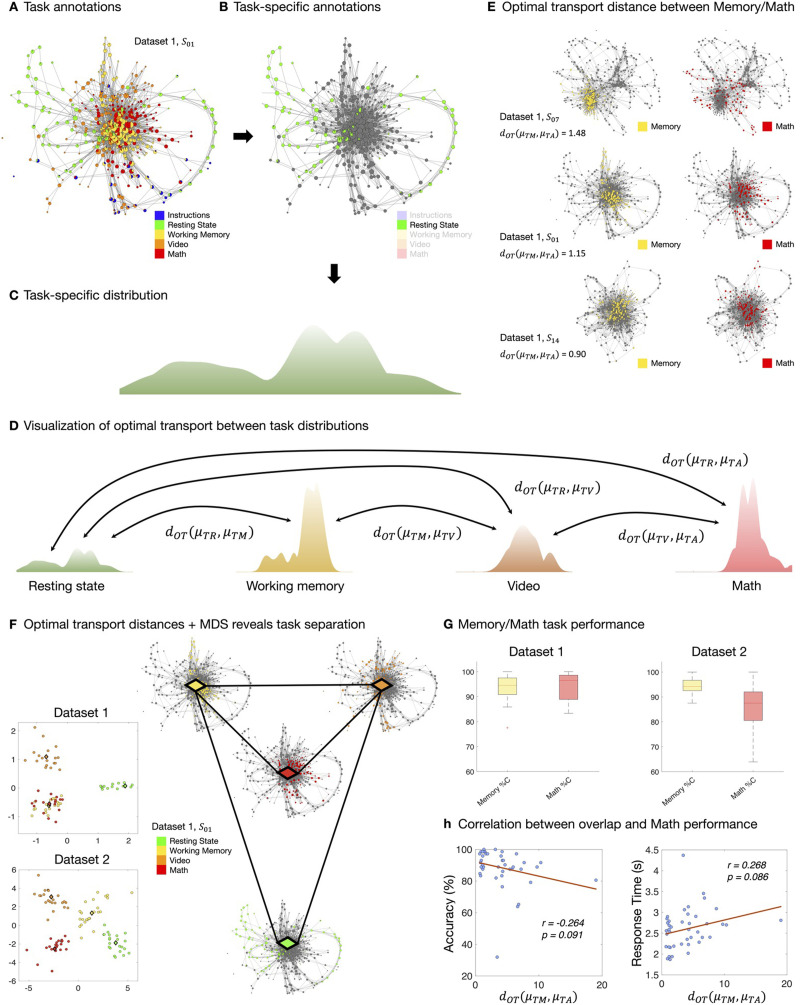
Quantifying pairwise similarity between annotations via optimal transport (OT). (A–C) Individual annotations can be isolated and normalized into probability distributions, which we can visualize as landscapes. (D) Annotations collapse data across time. Transitions in their spatial distribution can be quantified via OT distances. (E) These OT distances can be interpreted as follows: *S*_14_ has small *d*_*OT*_ because their memory and math annotations are close in the graph, whereas *S*_07_ has large *d*_*OT*_ because their memory and math annotations occupy different portions of the graph. We write *μ*_*TM*_, *μ*_*TA*_ for the task annotations corresponding to memory and arithmetic/math, respectively. (F) Because *d*_*OT*_ provides distances, the separation between annotations can be visualized via MDS projection. MDS plots of separation between task annotations across the two datasets are shown in the two panels. For the task annotations, the projection reveals a subtle difference in the shape graphs from Dataset 1 and 2, showing higher separation between math and memory annotations for Dataset 2 than for Dataset 1. This lack of overlap is unexpected, due to the recruitment of working memory during arithmetic tasks and may suggest poorer performance in the math task. (G) Separation between task annotations may be related to the higher separation between memory and math performance in Dataset 2 than in Dataset 1, as reported in the boxplots. (H) We further compute and report Pearson correlations between two quantities: (1) *d*_*OT*_ (*μ*_*TM*_, *μ*_*TA*_) between memory task and math/arithmetic task annotations, and (2) math performance in terms of accuracy and response time. These plots show that lower overlap between memory task and math task annotations is related to poorer math performance.

While the two mesoscale properties of shape graphs present critical insights about neurobehavior, they can still be thought of as first-order insights. Thus, even though these mesoscale properties inform about how individual task blocks are represented on the graph, they miss any putative second-order structure, for example, how well individual task blocks are separated from each other on the shape graphs. To better account for such second-order structures, we use tools from *optimal transport* theory (Peyré & Cuturi, 2019). The pie chart–based proportional annotation of a shape graph node means that each task block contributes a fraction (possibly zero) of the time points making up the node. After normalizing, each task block thus yields a probability distribution over the nodes of the graph. We compare the dissimilarities between these distributions using an OT distance *d*_*OT*_. Intuitively, task annotations correspond to different landforms making up the global landscape on which whole-brain dynamics occur during the CMP, and knowledge of pairwise distances between these landforms encodes the knowledge of the global structure of the landscape. Figure 3 further illustrates this construction on Datasets 1 and 2, where we additionally visualize interpolations between the shapes of the task landforms (Figure 3D).

The OT distances between tasks in the shape graphs possess nontrivial structure (Figure 3). For both Datasets 1 and 2, one-way ANOVA (Dataset 1: *F*(5,102) = 11.1, *p* = 1.4 · 10^−8^; Dataset 2: *F*(5,138) = 3.09, *p* = 0.011) and the Kruskal–Wallis test (Dataset 1: *H*(5) = 51.6, *p* = 6.5 · 10^−10^; Dataset 2: *H*(5) = 24.0, *p* < 0.001) revealed significant effects of task such that the whole-graph distributions of different task blocks were separated from each other.

To visualize between-task distances derived from OT, we used multidimensional scaling (Figure 3F). Across both datasets, tasks with low cognitive load (e.g., resting state and video watching) were well separated from those with putatively higher cognitive load (e.g., working memory and math). However, only in Dataset 2, math and working memory task blocks were also separated. Given the previous work that suggests a significant role of working memory during arithmetic, higher overlap between the two tasks was expected (Cragg & Gilmore, 2014; Raghubar et al., 2010). Lack of such overlap between working memory and math could indicate poor performance during arithmetic task. We verified this OT-generated observation by examining differences in behavioral performance during working memory and math tasks in both datasets. Although the performance during working memory was not different between datasets (*t* = −0.718, *p* = 0.477), there was a significant difference in math performance between datasets (*t* = 2.69, *p* = 0.010). Participants in Dataset 2 performed significantly worse in math than those in Dataset 1 (Figure 3G). Further, within Dataset 2, we observed a negative relation trending toward significance (Figure 3H) between OT-derived distance between memory and math and behavioral performance during math task as measured by accuracy (*r* = −0.264, *p* = 0.091) and response time (*r* = 0.269, *p* = 0.086). Thus, providing preliminary evidence in capturing behaviorally relevant interplay between working memory and arithmetic tasks using our NeuMapper framework.

### Anchoring Shape Graphs Into Known Cognitive Constructs

To anchor NeuMapper-generated shape graphs into known cognitive constructs and to potentially decode mental states revealed by the shape graphs, we annotated nodes using the NeuroSynth decoding database (Yarkoni et al., 2011). Each node of the shape graph was annotated by the strength of spatial cross-correlation between the brain configuration represented by that node and configurations for related cognitive topics from the NeuroSynth decoding database (Yarkoni et al., 2011). A similar decoding analysis using NeuroSynth topic terms has been previously performed for time-varying functional connectivity matrices (Gonzalez-Castillo et al., 2019).

Here, we first selected two topic terms from the NeuroSynth *v4-topics-50* database—task-positive (Topic 002) and task-negative (Topic 010)—to annotate shape graphs. We then compared “empirical” annotations of shape graph nodes based on NeuroSynth topic maps with annotations based on the expected task structure. We hypothesized that participants’ task performance would be higher whenever the empirical and expected annotations would match and that performance would be lower in case of a mismatch.

**Figure 4.**
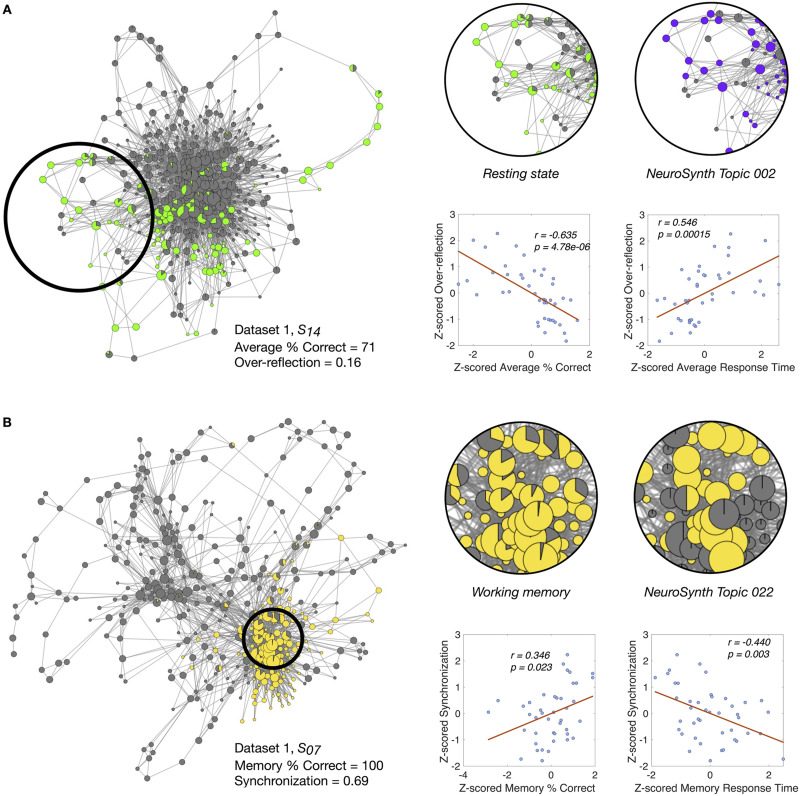
Novel insights from NeuroSynth-generated annotations. (A) We first computed an *overreflection* score to quantify how often whole-brain configurations visited during the resting state were more similar to meta-analytic brain configurations generally associated with task-related cognition (i.e., Topic 002). Here we show the shape graph for a poor performer from Dataset 1, highlighting an example where the participant may have been thinking excessively of a task when the experimental instruction was to remain at rest. The nodes are colored by the experimental rest condition and the NeuroSynth-decoded task-positive topic annotations. The corresponding plots show the Pearson correlation between the (*z*-scored) overreflection score and average task accuracy/response time. (B) We then computed a “synchronization” score to quantify how often brain configurations visited during the working memory task were more similar to the meta-analytic brain configuration generally associated with working memory. Here we show the shape graph for a good performer from Dataset 1, highlighting the annotation overlap captured by the synchronization score. The nodes are colored by the experimental working memory task structure and NeuroSynth-decoded working memory topic annotations (i.e., Topic 022). The corresponding plots show the Pearson correlation between the (*z*-scored) synchronization score and accuracy/response time for the working memory task.

Interestingly, we observed that eliciting brain configurations that are more similar to the task-positive topic model during the expected resting-state blocks (i.e., a mismatch) was significantly negatively correlated with the percentage of correct responses (averaged across tasks; *r* = −0.635, *p* = 4.78 · 10^−6^) and significantly positively correlated with average response time (averaged across tasks; *r* = 0.546, *p* = 0.00015) (Figure 4A). These findings suggest that perhaps this negative relation is associated with putative *overreflection* about tasks during periods of rest (i.e., when the participants are instructed to let their minds wander). Note that we did not find any significant correlations in the reverse case, that is, eliciting brain configurations akin to task-negative topic model during nonresting-state task blocks.

Next, we computed a *synchronization* score by counting the proportion of nodes where the empirical task-positive annotation coincided with the expected task (M, V, A) blocks and the task-negative annotation coincided with the expected rest (R) block. As hypothesized, we observed a significant correlation between the synchronization score and the average accuracy (*r* = 0.422, *p* = 0.005) and response time (*r* = 0.474, *p* = 0.001). To go further beyond the task-positive topic model, we then asked ourselves if specific topic models corresponding to the tasks (M, V, A) could be related to performance in each individual task. Because the n-back working memory paradigm has been widely used in the literature and has been highly sampled in the NeuroSynth database (i.e., Topic 022 is associated with 933 studies), we limited ourselves to the working memory task block. Specifically, using a specific topic model for the working memory task (Topic 022), we examined whether performance in the working memory blocks is associated with eliciting brain configurations that are more similar to the meta-analytic brain configurations generally related with working memory. Here, we again found that having a higher match between empirical and expected task annotations—that is, memory (Topic 022) annotations predicted by NeuroSynth in nodes that were also annotated with the memory experimental task label—was significantly positively correlated with the percentage of correct responses for the memory task (*r* = 0.346, *p* = 0.023) and significantly negatively correlated with response time for the memory task (*r* = −0.440, *p* = 0.003) (Figure 4B). In other words, this finding suggests that an individual’s performance tends to increase when the participants duly engage the task-specific brain circuits while performing the task. This results also amplifies the putative role that NeuMapper can play in decoding mental states.

## DISCUSSION

We present a fast, end-to-end computational framework that incorporates and extends the Mapper algorithm—a powerful manifold learning technique within the suite of methods provided by the field of TDA (Lum et al., 2013; Rabadán & Blumberg, 2019; Singh et al., 2007)—by integrating novel algorithmic contributions as well as downstream processing techniques for capturing second-order mesoscale structure and meta-analysis guided inference. We used our NeuMapper framework to study multiple functional neuroimaging datasets where participants engaged in a CMP simulating ongoing cognition. Our adaptations of methods for approximating nonlinear geometry revealed interesting topological structure in our datasets from the outset and our heuristics for parameter selection enabled efficient discovery of meaningful scales at which to observe various types of community structure in the data. We introduced tools from OT theory (Peyré & Cuturi, 2019) to further understand the second-order structure of groupings of data points as delineated by experiment design, and also introduced the use of NeuroSynth-based meta-analyses (Yarkoni et al., 2011) to enrich our shape graphs with additional semantic meaning. Finally, we translated these computational methods into markers of individual differences in how the brain adapts to stimuli during ongoing cognition. In summary, we provide a validated computational pipeline for neuroimaging data that can be easily used by researchers and clinicians for interactive data representation with simultaneous access to quantitative insights.

The success of approaches based on dynamic methods for translating patterns of ongoing cognition into meaningful cognitive states has led to a burgeoning landscape of computational methodologies for functional neuroimaging data (Bassett et al., 2011; Bullmore & Sporns, 2009; Calhoun et al., 2014; Lurie et al., 2020; Preti et al., 2017; Sporns, 2011; Tagliazucchi et al., 2012; Turk-Browne, 2013; van der Meer et al., n.d.; Vidaurre et al., 2017). In contrast to the dynamic functional connectivity approaches that estimate interregional coactivity over time, topology/geometry processing techniques such as Mapper instead use whole-brain activation patterns to infer the shape of an underlying landscape of brain activity (Geniesse et al., 2019; Saggar et al., 2018). By enriching this inferred landscape with annotations coming from experimental task block labels or meta-analysis based decoding (Yarkoni et al., 2011) and subsequently capturing second-order interactions across groups of annotations—both experimentally based and decoded—we show that it is possible to use TDA methods to both infer dynamics and obtain a semantic segmentation of the underlying state space. This exploration of second-order interactions is concurrent with the development of related strategies for higher order dynamic connectivity (Faskowitz et al., 2020; Owen et al., 2019). Overall, we corroborate and extend prior findings (Saggar et al., 2018) on the relation between task performance and quantitative measures of shape graphs, and further contribute to the computational methodologies for understanding the brain’s dynamic adaptations to external stimuli.

Our framework extends the traditional Mapper approach without discarding any of its key properties, and the modular organization of our pipeline suggests replacements based on user preference. For example, numerous works (Cunningham & Yu, 2014; Nichols et al., 2017) have found evidence to suggest that brain activity is fundamentally low dimensional, and that a linear technique such as principal component analysis (PCA) potentially captures this activity along interpretable axes (Shine et al., 2019a; Shine et al., 2019b). Our framework could be freely used to this end, by simply replacing the *k*NN graph construction with a PCA projection. At the same time, depending on the amount of variance to be explained, one may need a moderate to high number of projection dimensions when carrying out PCA. More generally, dimension reduction techniques tend to preserve more information as the embedding dimension increases (see Figure 1C, for an example). Runtime comparisons showed that our intrinsic binning approach tended to be much faster than existing open-source Mapper implementations such as Giotto-Mapper and KeplerMapper when higher embedding dimensions were used, suggesting that our methods could be incorporated as modules within such frameworks (Tauzin et al., 2021; van Veen et al., 2019). We note also that techniques for high-dimensional information retrieval and clustering (Agrawal et al., 2005; Beyer et al., 1998; Hinneburg & Keim, 1999; Indyk & Motwani, 1998; Radovanović et al., 2010) could potentially be used to develop a unified computational framework for the binning stage of the Mapper algorithm that scales well with dimension, and use this work to invite future progress in developing Mapper applications that do not use low-dimensional embeddings at the outset. Recent works have fused graph neural network techniques with Mapper to consume graph-structured data as input and return meaningful embeddings (Bodnar et al., 2020), and such a module could be easily inserted between the reciprocal *k*NN and intrinsic binning steps in our framework to take advantage of the representational power of neural networks. Along the lines of hardware-driven scalability, Mapper Interactive (Zhou et al., n.d.) provides state-of-the-art GPU implementations of the Mapper algorithm for embedding dimensions 1 and 2, and our method could be adapted to fit into such a pipeline. In addition to such scalability improvements, semiautomated mesoscale network structure analysis is a fundamental aspect of our pipeline, and we have demonstrated multiple ways in which a user can supply annotations (i.e., based on task structure or NeuroSynth meta-analyses) to yield quantitative results from data. Combining these modules with the functionality of existing open-source Mapper implementations may be crucial in obtaining new insights via geometric and TDA on consortium-sized data (Marek et al., 2020).

Toward moving Mapper analysis from exploratory to quantitative, we introduced OT techniques that naturally delineate the overlapping categorical labels of shape graph nodes and quantify the dissimilarity between categories from the perspective of the shape graph. From an interesting dual perspective, the shape graphs themselves may thus be viewed as filters through which to quantify the overall landscape of different cognitive constructs, hence lending a *Mapper of Mappers* theme to our contributions. Our use of NeuroSynth-based decoding provided a new angle on obtaining *learned* categorical labels and introduced a novel study of *semantically-aware TDA* in analogy with the semantic segmentation approaches in modern deep-learning based computer vision (Qi et al., 2017). Our developments suggest that it may be helpful to view Mapper as less of a fixed, immutable algorithm, and more of a *philosophy* that may be woven into alternative and diverse computational pipelines.

With regards to neuroimaging studies, an important issue that is not directly addressed in this work and requires future effort is to estimate the minimum amount of data required per individual for stable estimation of Mapper-generated shape graphs. In this and previous works using Mapper, we used 20–30 min of task fMRI data per individual. While access to large quantities of artifact-free data (Gordon et al., 2017) would be ideal for any computational method, for clinical populations it is often more practical to aggregate data across sites and studies (Saggar & Uddin, 2019). In the current work, we carried out two forms of aggregation: (1) Dataset 2 comprised two runs for each subject that we aggregated before computing shape graphs, and (2) shape graph properties such as modularity were aggregated across both datasets when reporting relations to behavior. Note that Datasets 1 and 2 were obtained at different sites and under different acquisition parameters, including scanner strengths and repetition times. Because our framework provided successful inference on the aggregated shape graph properties, we suggest that future work could consider further aggregations of shape graph properties for data collected under more variable acquisition parameters to fully test any limitations of our method. We also note that in related work, Saggar et al. (2021) applied the traditional Mapper approach to data from 100 unrelated subjects in the Human Connectome Project combined over four 15-min runs. Altogether, these observations suggest that Mapper-based frameworks may be well suited for data aggregation across runs and that shape graph properties may be aggregated across datasets acquired under different scanning parameters.

While we focused on some of the most salient aspects of neuroimaging-specific Mapper design, there were certain choices that we left for future work. In our NeuMapper filter design, the landmarks obtained via farthest point sampling do not have any particular biological relevance. However, it is possible to augment this step by choosing landmarks using some additional criterion, for example, by averaging over a proportion of frames to define a “baseline” state for each task (Duman et al., 2019; Khambhati et al., 2018). In particular, for resting-state studies, an interesting possibility in our NeuMapper design could be to choose a single base point in the *k*NN graph representing a baseline state, and then filter the remaining graph vertices by a one-dimensional number: the distance to the base point (Chowdhury et al., 2020). This one-dimensional filter setting allows the import of the statistical guarantees provided by the seminal work of Carriere et al. (2018) and should be carried out in future work. Further down the Mapper pipeline, the partial clustering step is itself a specialized clustering problem for which Belchi et al. (2020) have provided theoretical guarantees as well as practical frameworks utilizing *k*-fold cross-validation. For expediency, we performed quick initial checks using hierarchical and nonhierarchical clustering techniques (James et al., 2000) such as average linkage, complete linkage, DBSCAN, and HDBSCAN before settling on a simplistic choice of single linkage with a fixed cutoff parameter. Future work could look into invoking neurobiologically motivated clustering techniques along with additional cross-validation (Belchi et al., 2020).

As shown with CMP datasets, the shape graph representation of functional brain activity converts high-dimensional neural activity recordings into a landscape on which we can study whole-brain dynamics driven by the processes underlying cognitive states, and ultimately relate these analyses to multidimensional behavioral measures and clinical outcomes. A critical next step will be to demonstrate how our NeuMapper framework can be applied in unconstrained resting-state paradigms, where there is no known ground truth of the internal cognitive state of each participant. Our demonstration of NeuroSynth-based decoding of underlying cognitive states, following Gonzalez-Castillo et al. (2019), provided strong quantitative suggestion that such an approach could be viable and successful. While we did not test our approach on resting-state or naturalistic fMRI data, we note that the standard Mapper approach (extrinsic 2-D binning) has recently been applied (Saggar et al., 2021) to 5 hours of resting-state fMRI data from the Midnight Scan Club dataset (Gordon et al., 2017). There the authors observed highly subject-specific shape graphs with a central “basin of attraction” surrounded by peripheral areas having distinct network configurations. Based on these results, we expect that our approach will extend nicely to resting-state and naturalistic paradigms. Moreover, the scalability of our approach will allow us to explore some of the consortium-level naturalistic datasets that are increasingly becoming available.

While designing our NeuMapper framework, we paid particular attention to potential scalability issues when working with the consortium-size datasets needed to reduce statistical error in brain-behavior association studies (Marek et al., 2020). Given a fixed set of parameters and assuming the use of the nonlinear reciprocal *k*NN graph construction, the most expensive computation in our framework comprises a fixed number of calls to the *O*((|*V*| + |*E*|) log(|*V*|)) Dijkstra algorithm. For standard fMRI datasets of individual participants with up to several thousand frames, this computation can be carried out within seconds on standard hardware. For group-level analysis where datasets may be concatenated to contain up to several million frames (e.g., for datasets such as the Human Connectome Project), we may utilize GPU libraries such as cuGraph or nvGRAPH to carry out such computations in a few hours. The remaining steps of the NeuMapper pipeline—namely parameter selection and post hoc computations—are easily parallelized, for example, via the implementation that we used in this work. Future work should investigate large-scale application of NeuMapper to consortium-size datasets in order to generate a population-level “template” landscape on which we can map and study the dynamics of whole-brain activation during cognition. Because shape graphs may be of different sizes, this requires solving a node-correspondence problem. This can potentially be resolved using our recently developed extensions of OT theory for scalable generation of correspondences (Chowdhury & Mémoli, 2019; Chowdhury & Needham, 2020, 2021). Looking forward, our OT based approach could allow researchers to carefully parse population-level heterogeneity in the data, for example, by applying PCA on the space of shape graphs (Chowdhury & Needham, 2020).

Lastly, we expect that a powerful use case of our NeuMapper framework will be in the setting of multimodal neuroimaging data analysis, where different types and scales of information that are uniquely captured by different neuroimaging modalities are combined into an overall representation that conveys more information than any individual modality. For example, analyses of the inherent temporal structure and dynamical properties measured by fMRI data could be augmented by high-resolution anatomical information provided by diffusion tract imaging. Combining these different information-rich sources of data into unified and individual-specific descriptions and signatures of brain activity could better capture individual differences in behaviorally relevant dynamics and in turn could improve the prospects of precision medicine.

In summary, we provide a computationally scalable, biologically anchored, and downstream analysis-friendly Mapper framework for application in the empirical sciences in general and neurosciences in particular.

## METHODS

### Data Acquisition

#### Dataset 1: Continuous multitask paradigm.

In this study, we utilized a previously collected continuous multitask fMRI dataset to develop our framework, and we acquired a second dataset using a similar paradigm but at a faster temporal resolution to validate our approach. The first dataset was originally collected by Gonzalez-Castillo et al. (2015), using a CMP. We retrieved the data from the XNAT Central public repository (https://central.xnat.org; Project ID: FCStateClassif). The dataset contained de-identified fMRI and behavioral data from 18 participants who completed the CMP experiments as part of the original study (Gonzalez-Castillo et al., 2015). Informed consent was obtained from all participants, and the local Institutional Review Board of the National Institute of Mental Health in Bethesda, MD, reviewed and approved the CMP data collection.

Details about the experimental paradigm are described elsewhere (Gonzalez-Castillo et al., 2015). Briefly, participants were scanned continuously for ∼25 min while performing four different cognitive tasks. Each task was presented for two separate 180-s blocks, with each task block being preceded by a 12-s instruction period. The order of task blocks was randomized such that each task was always preceded and followed by a different task. The same random ordering of tasks was used for all participants. The four cognitive tasks were (1) Rest (R), where participants were instructed to fixate on a crosshair in the center of the screen and let their mind wander; (2) Working Memory (M), where participants were presented with a continuous sequence of individual geometric shapes and were instructed to press a button when the current shape had also appeared two shapes prior (2-back design); (3) Math/Arithmetic (A), where participants were presented with simple arithmetic operations, involving three numbers between 1 and 10 and two operands (either addition or subtraction)—operations remained on the screen for 4 s, and successive trials were separated by a blank screen that appeared for 1 s, yielding a total of 36 operations per each 180-s block—and (4) Video (V), where participants watched a video of a fish tank from a single point of view with different types of fish swimming into an out of the frame. Participants were instructed to press a button when a red crosshair appeared on a clown fish and another when it appeared on any other type of fish. These targets appeared for 200 ms with a total of 16 targets during each of the 180-s blocks.

The fMRI data were acquired on a Siemens 7 Tesla MRI scanner equipped with a 32-channel head coil using a whole-brain echo planar imaging (EPI) sequence (repetition time [TR] = 1.5 s, echo time [TE] = 25 ms, and voxel size = 2 mm isotropic). A total of 1,017 volumes were acquired while participants performed the CMP.

Functional and anatomical MR images were preprocessed using the Configurable Pipeline for the Analysis of Connectomes (C-PAC version 0.3.4; https://fcp-indi.github.io/docs/user/index.html). Details about this processing are provided elsewhere (Saggar et al., 2018). Briefly, the fMRI data preprocessing steps included ANTS registration into MNI152 space, slice timing correction, motion correction, skull stripping, grand mean scaling, spatial smoothing (FWHM of 4 mm), and temporal band-pass filtering (0.009 Hz < f < 0.08 Hz). For each voxel, nuisance signal correction was performed by regressing out linear and quadratic trends, physiological noise (white matter and cerebrospinal fluid), motion-related noise (three translational and three rotational head-motion parameters) using the Volterra expansion (Friston et al., 1996) (i.e., six parameters, their temporal derivatives, and each of these values squared), and residual signal unrelated to neural activity extracted using the CompCor algorithm (Behzadi et al., 2007) (i.e., five principal components derived from noise regions in which the time series data were unlikely to be modulated by neural activity). The resulting data were brought to 3-mm MNI space, and the mean time series was extracted from 375 predefined regions of interest (ROIs) using the Shine et al. (2016) atlas. The atlas includes 333 cortical regions from the Gordon et al. (2016) atlas, 14 subcortical regions from the Harvard-Oxford subcortical atlas, and 28 cerebellar regions from the SUIT atlas (Diedrichsen et al., 2009). Before running Mapper, the preprocessed ROI time series data were converted to *z*-scores, and individual ROIs with zero variance were excluded.

The behavioral data included both responses and reaction times for working memory, math, and video tasks. Participants were instructed to respond as quickly and accurately as possible with only one response per question. Behavior scores including the percent correct, percent missed, and response times for Working Memory (M), Math/Arithmetic (A), and Video (V) tasks were computed for each participant.

#### Dataset 2: Continuous multitask experiment.

We collected the second fMRI dataset at a faster temporal resolution using a modified CMP (Gonzalez-Castillo et al., 2015) as part of a simultaneous EEG and MRI functional neuroimaging (SEMFNI) study. The raw data includes fMRI scans and behavioral scores collected for 32 participants who completed two separate continuous multitask runs within a single scanning session, as part of the SEMFNI study. Informed consent was obtained from all participants, and the Stanford University Institutional Review Board reviewed and approved the SEMFNI data collection.

Briefly, the paradigm used by the continuous multitask experiment was adapted from the original CMP (Gonzalez-Castillo et al., 2015) so that data could be collected for two separate runs over the course of a single ∼30-min session. For each run, participants were scanned continuously for ∼15 min while performing four different cognitive tasks. Each task was presented for two separate 90-s blocks, with each task block being preceded by a 12-s instruction period. The order of task blocks presented during each run was randomized such that each task was always preceded and followed by a different task. The same random ordering of tasks was used for all participants and for both runs. The randomized order of trials presented during each task block was modified from the original paradigm (Gonzalez-Castillo et al., 2015) such that the trials presented during the first run were different than the trials presented during the second run (i.e., trials from the first 90 s of each 180-s task block in the original paradigm were used for the first run of the modified paradigm, trials from the second half of each 180-s task block in the original paradigm were used for the second run of the modified paradigm).

The fMRI data were acquired on a modified Siemens Skyra 3 Tesla MRI scanner equipped with a 32-channel head coil using a whole-brain EPI sequence (repetition time [TR] = 750 ms, echo time [TE] = 30 ms, and voxel size = 2 mm isotropic). A multiband acceleration factor of 8 was used to increase temporal resolution. A total of 2,160 volumes were acquired over two separate runs. The 1,080 volumes acquired during each run were preprocessed separately.

Functional and anatomical MR images were preprocessed using fMRIPrep (version 1.5.4; https://fmriprep.readthedocs.io) (Esteban et al., 2018a, 2018b). Before running fMRIPrep, bias correction was performed on individual anatomical MR images using FMRIB’s Automated Segmentation Tool. Individual functional MR images and the bias-corrected anatomical MR images were transformed into MNI152 space using FSL’s MNI ICBM 152 nonlinear 6th Generation Asymmetric Average Brain Stereotaxic Registration Model (Evans et al., 2012). The fMRI data preprocessing steps included skull stripping, head motion and susceptibility distortion correction, boundary-based registration, and confound collection. After running fMRIPrep, motion censoring, demeaning, detrending, nuisance signal correction, and temporal filtering were performed in Matlab. Here, we only considered voxels within the gray matter. The brain masks provided by fMRIPrep were brought to 2-mm MNI space (in version 1.5.4 the provided brain masks retain the original 1-mm pixel dimensions), and these 3-D spatial masks were then used to extract the gray-matter voxel time series from the preprocessed 4-D fMRI data. The resulting 4-D fMRI signals were then transformed into a 2-D matrix. The first 10 frames (i.e., nonsteady state) of data were discarded, and individual time frames with high framewise displacement (FD >0.5 mm) were annotated as motion outliers. The data corresponding to these frames were replaced with NaN values, such that these frames were ignored during subsequent processing steps. After motion censoring, the voxel-wise data were demeaned, and linear trends were removed. For each voxel, nuisance signal correction was then performed by regressing out physiological noise (white matter and cerebrospinal fluid) and motion-related noise (three translational and three rotational head-motion parameters) using the Volterra expansion (Friston et al., 1996) of the two physiological signals and six motion parameters (i.e., eight parameters, their temporal derivatives, and each of these values squared). Linear interpolation was applied to the residual voxel signals to smooth over any missing data corresponding to high-motion frames, and temporal band-pass filtering (0.009 Hz < f < 0.08 Hz) was performed. The mean time series was extracted from 375 predefined ROIs using the Shine et al. (2016) atlas. The atlas includes 333 cortical regions from the Gordon et al. (2016) atlas, 14 subcortical regions from the Harvard-Oxford subcortical atlas, and 28 cerebellar regions from the SUIT atlas (Diedrichsen et al., 2009). Before running Mapper, individual runs were concatenated together, and individual ROIs with zero variance were excluded.

Behavioral data was collected during each run, including responses and reaction times for the different tasks. Participants were instructed to respond as quickly and accurately as possible with only one response per question. Behavior scores including the percent correct (%C), percent missed, and reaction times for Working Memory (M), Math/Arithmetic (A), and Video (V) tasks were computed (and averaged across runs) for each participant.

Note that one participant’s data were excluded from preprocessing altogether due to inconsistent data acquisition (i.e., too many volumes were collected during the second run due to a technical issue), one participant was excluded from our final analysis due to excessive in-scanner head motion (i.e., more than 10% of the acquired datapoints had framewise displacement above 0.5 mm), and five participants were excluded from the final analysis because their average %Correct scores were substantially lower than those of the other participants. Specifically, after plotting all average %Correct scores on a histogram with bins of width 10, the scores of these five participants comprised a left tail (i.e., very poor performance) that was clearly separated from the scores of all other participants. At the final stage, Dataset 2 included 25 participants. Overall, this yielded *n* = 43 participants across both datasets.

### The NeuMapper Framework

#### Geometric and TDA for neuroimaging data.

Consider an *n* × *p* data matrix *X* where the columns correspond to voxels or brain ROIs and the rows correspond to acquisitions of whole-brain activation patterns. Standard neuroimaging techniques such as functional connectivity (FC) or dynamic functional connectivity (dFC) work on the columns of the data matrix to produce one or more *p* × *p* correlation matrices. While such techniques capture the coactivation of brain regions in response to external stimuli, an alternative approach is to study *n* × *n* matrices of distances between acquisitions to understand the overall geometry of the states traversed by the brain during the experiment. Examples such as Anscombe’s quartet (Anscombe, 1973) and related generalizations (Matejka & Fitzmaurice, 2017) show that studying this underlying geometry may provide strictly complementary insights, and in our work we emphasize the importance of studying this geometry of brain states.

Each of our data matrices had rows labeled by the tasks in the experiment design (Rest, R; Memory, M; Video, V; and Math/Arithmetic, A). To obtain a coarse understanding of the high-dimensional geometry of the data, we first applied a variety of linear and nonlinear dimension reduction methods (Van Der Maaten et al., 2009) to data from a single subject (*S*_01_ from Dataset 1) and visualized the results in simple 3-D plots. The full list of methods and plots is provided in Supporting Information Figure S8. Of these methods, the linear approaches, for example, PCA, attempt to find linear combinations of features that best explain the data. Such approaches, while useful, do not consider the intrinsic geometry of data. In contrast, nonlinear approaches attempt to capture intrinsic geometry by considering the local neighborhood of each data point, as determined by a *k*-nearest neighbor (*k*NN) graph built on the data. While promising for capturing the geometry of brain states, such approaches work best when the data is sampled both uniformly and without noise from its underlying distribution, and are otherwise prone to a type of error called *topological instability* (Balasubramanian & Schwartz, 2002). Because fMRI data are inherently noisy and suffer from sampling variability, we expect that this error would be unavoidable for standard methods and thus resort to specialized techniques that capture intrinsic geometry while mitigating such errors due to noise.

One plausible solution to the topological instability problem is to use techniques from TDA, namely, the Mapper algorithm. The standard Mapper approach initially applies a *d*-dimensional projection to the data matrix (*filtering*), and then constructs a *d*-dimensional grid with overlapping cells to cover the projected data points (*binning*). A clustering scheme is then applied to refine each cell (*partial clustering*), and finally a graph is constructed with the refined cells as nodes, and edges between nodes if the corresponding cells share one or more data points. Notably, the partial clustering scheme operates on the native high-dimensional embedding of the points in each cell, and thus avoids some of the information loss due to projection. Specifically, low-dimensional projection tends to erroneously collapse points close together, and partial clustering attempts to reverse this collapse. Thus, a way to address the topological instability of *k*NN-based approaches is to fuse together the partial clustering step from the Mapper algorithm.

However, there is still a caveat that information loss is inevitable when representing data that is natively on the order of several hundred to thousands of dimensions in just two or three dimensions. Given that the final output of Mapper is a combinatorial graph, there is a surprising lack of methods in the existing literature that implicitly map the data matrix *X* into the output graph without incurring the information loss from low-dimensional projection. Here, we propose such an implicit method that moves away from low-dimensional projection and operates directly at the level of distance matrices. The key insight is that we can obtain the topological stabilization properties of Mapper by operating directly on the native high-dimensional metric *D* and any transformation *D*′ thereof without explicitly constructing a low-dimensional embedding.

#### Reciprocal kNN.

To gain access to the intrinsic geometry of the high-dimensional data, our constructions leverage a particular type of *k*NN graph and the matrix of geodesic distances on this graph. First, we fix a choice of metric for our dataset *X* (in our case, the *L*^1^ metric, also known as the Manhattan distance) and use this to build an *n* × *n* matrix *D* of pairwise distances between the rows of *X*. We choose *L*^1^ over the more standard *L*^2^ (Euclidean) metric due to higher effectiveness for nearest neighbor searches in high dimensions (Aggarwal et al., 2001). Next, for each row *x*, we select the top-*k* nearest neighbors and call this set *NN*_*k*_(*x*). Then we build a graph *G* where the node set is indexed by *X*, and an edge (*x*_*i*_, *x*_*j*_) is added whenever *x*_*i*_ ∈ *NN*_*k*_(*x*_*j*_) and *x*_*j*_ ∈ *NN*_*k*_(*x*_*i*_). This final condition of creating edges by a symmetric criterion is referred to as *reciprocal kNN*. Notice that these operations can be easily implemented using sort operations. We finally define a new metric matrix *D*′ by taking the shortest path distances (i.e., geodesic distances) on *G*. Note that this can be implemented in *O*(|*V*|^3^) time using a Floyd–Warshall algorithm, although the next section shows that we can alternatively utilize a small number of calls to the *O*((|*V*| + |*E*|) log(|*V*|)) Dijkstra algorithm.

The choice of *reciprocal k*NN is a crucial aspect of our method, as it helps stabilize the noise levels of fMRI data by pruning connections across areas with different local data density (Qin et al., 2011). Supporting Information Figure S6 contains a toy example illustrating how reciprocal *k*NN reduces “false” connections compared to standard *k*NN. Additionally, in Supporting Information Figure S8 we show for an exemplar subject in Dataset 1 that replacing the *k*NN construction in a standard nonlinear dimension reduction method with a reciprocal *k*NN construction yields a low-dimensional embedding where data points associated to different tasks form distinctive topological structures. We also note that *k*NN constructions have been used before in Mapper applications (Duponchel, 2018), although in such applications the *k*NN graph construction has been followed by a low-dimensional projection via a graph drawing algorithm. In contrast, we use a tall, skinny submatrix of the matrix *D*′ with columns corresponding to landmarks chosen via the procedure described next.

Finally, we note that the dimensionality of the data has an important effect on the computation of nearest neighbors. While techniques such as kd-trees can be very effective for nearest neighbor queries in dimensions below 10, for dimensions *p* > 10 it may be preferable to compute nearest neighbors via a linear scan which runs in in *O*(*np*) time (Weber et al., 1998). To obtain sublinear dependence on *n*, it is possible to use techniques such as locality sensitive hashing (Indyk & Motwani, 1998), although this approach is not addressed in the current work. In Supporting Information Figure S7 we show that the runtime of our method initially scales linearly with data dimension before flattening and showing logarithmic dependence. We also note that when applying our method to voxel-level fMRI data, where *p* is in the order of hundreds of thousands, one may use scalable approximate nearest neighbor methods (Dong et al., 2011).

Thus far, we have replaced the filtering step, that is, the low-dimensional projection, of the standard Mapper algorithm by a technique for obtaining a pairwise distance matrix *D*′ that encodes the intrinsic geometry of the data. Next, we carry out a sampling procedure that obtains landmarks on the data for use in a binning step while assuming access only to *D*′.

#### Farthest point sampling (FPS).

FPS is a greedy algorithm for landmarking data (Gonzalez, 1985). In this algorithm, one starts with a seed point *x*_0_ ∈ *X*. Then one chooses *x*_1_ to be a point that maximizes the *D*′ distance to *x*_0_, possibly with some arbitrary tie breaking between equidistant points. Next one chooses *x*_2_ to be a point that maximizes the distance to {*x*_0_, *x*_1_}, where the latter is defined as min(*D*′(*x*_2_, *x*_0_), *D*′(*x*_2_, *x*_1_)). The algorithm proceeds in this manner until *x*_*r*−1_ has been chosen, where *r* is a user-specified *resolution* parameter. We utilized the Matlab implementation provided in (De Silva & Perry, 2003). Note also that each step requires only a call to Dijkstra’s algorithm for a total of *r* calls, as well as some max and min operations. The final output of this step is a set of landmark points {*x*_0_, *x*_1_, …, *x*_*r*−1_} and a real number *ϵ* corresponding to the maximal distance from any point in *X* to its closest landmark point according to *D*′.

Toward reproducibility, we attempted to minimize randomness during landmark selection in two ways. First, while FPS typically begins with the random selection of an initial seed point, we instead define the seed point to always be the first row of *X*. This ensures that landmark selection is at least reproducible given the same input matrix *X*. Second, while the sequence of landmarks visited during FPS depends heavily on the choice of initial seed point, the following argument shows that the output of applying FPS to *D*′ is minimally affected by randomness in the seed point. First, FPS achieves a 2-approximation of the optimal locations for placing landmarks (also known in the literature as the metric *k*-center problem) (Gonzalez, 1985). Next, by appealing to this result and additional results from metric geometry (Burago et al., 2001, Example 7.3.11 and Corollary 7.3.28), any two sets of samples obtained by FPS yield distance matrices that are guaranteed to approximate the input distance matrix *D*′ up to a small multiplicative factor. This approximation guarantee in turn ensures that landmark selection is minimally affected by randomness, and the approximated distance matrix converges to *D*′ as the number of landmarks approaches the number of data points.

#### Intrinsic binning.

Having constructed landmarks, we now partition the data into *r* overlapping bins as follows: for each landmark *x*_*i*_, define

$$B_{i}≔\left\{x:D^{\prime }\left(x_{i},x\right)\leq 4\epsilon \cdot \frac{g}{100}\right\},$$

where *g* is a *gain* parameter that controls the level of overlap. The choice of *ϵg*/25 is set up for the following scenario: suppose *x*_*i*_, *x*_*j*_ are two landmarks satisfying *D*′(*x*_*i*_, *x*_*j*_) = 2*ϵ* and *p* is a point such that *D*′(*x*_*i*_, *p*) = *ϵ* = *D*′(*p*, *x*_*j*_). Then a gain of 50 (interpreted as 50%) allows the inclusion *x*_*j*_ ∈ *B*_*i*_. We set the minimum value of *g* to 25, which ensures that the collection of bins *B*_*i*_ fully covers *X*. We refer to this procedure of binning points using landmarks and the intrinsic metric *D*′ as *intrinsic binning*. In contrast, the standard Mapper algorithm uses a *d*-dimensional grid with overlapping cells that fully covers a *d*-dimensional projection of *X*, and we refer to this approach as *extrinsic binning* due to its use of the ambient space ℝ^*d*^. Note that *d*-dimensional cubes tend to be mostly empty when *d* is large, and hence the extrinsic binning approach becomes increasingly wasteful and computationally expensive as *d* increases. Further analysis of this extrinsic binning procedure is carried out in Dłotko (2019), where additionally a linear variant of intrinsic binning was utilized.

A property of this intrinsic binning procedure over binning using *d*-dimensional cubes is that it is data driven, and is grounded in techniques commonly used in TDA methods (Giusti et al., 2016). The resolution and gain parameters used above are analogous to those used in the conventional Mapper algorithm. We note that controlling gain via a single parameter is not necessarily optimal, as our data is unlikely to be uniformly sampled. Techniques such as UMAP (McInnes et al., 2018) suggest that one may instead carry out local computations around each landmark to set adaptive gain values *g*_*i*_ for each *B*_*i*_ that better reflect the data distribution. However, invoking such a method requires much more additional mathematical machinery, and we leave such extensions to future work.

To complete the description of the intrinsic binning procedure, we consider the case where *G* is not connected. In some techniques utilizing *k*NN graphs, one often proceeds by dropping all but the largest connected component (Van Der Maaten et al., 2009), which directly causes information loss. In our setting, however, we simply reallocate the number of landmark points to use for each connected component. Specifically, we allocate

$$\text{ceiling}\left(r\cdot \frac{\text{\#}\;\text{nodes}\;\text{in}\;\text{component}}{\text{total}\;\#\;\text{nodes}}\right)$$

landmark points to each connected component and perform binning for each component individually as above.

A different strategy that we also tried was to form a connected graph by augmenting edges with exponentially penalized weights, as described in (Bayá & Granitto, 2011). However, the binning strategy used above yielded greater signal and statistical power in downstream analysis.

Toward scalability, we next consider computation times of intrinsic versus extrinsic binning. In our tests, standard open-source Mapper implementations (KeplerMapper; van Veen et al., 2019; and Giotto-Mapper; Tauzin et al., 2021) suffered from significant slowdowns (Figure 1B) when carrying out the extrinsic binning procedure of the traditional Mapper approach after projecting data to spaces of four or more dimensions. A natural question then becomes: how many dimensions are needed to represent data? Intuitively, more dimensions are better for preserving information. In our case, to use existing open-source Mapper implementations after constructing a reciprocal *k*NN graph and computing graph geodesic distances, the easiest approach would be to apply an MDS projection in analogy with the Isomap algorithm for dimension reduction (Tenenbaum et al., 2000). We observed (Figure 1C) that the Pearson correlation between graph geodesic distances and distances after MDS projection increased with the dimension of the target space. Specifically, we needed 4- and 8-dimensional projections, respectively, to achieve correlation values exceeding 0.85 and 0.9. This suggests that information loss would be reduced by using a larger projection dimension, which unfortunately runs into the issue of slow runtime (Figure 1B). In contrast, once a matrix of distances *D*′ has been provided, our projection-free intrinsic binning approach can be naively implemented in *O*(*nr*) operations, where *n* is the number of data points and *r* is the number of landmarks. In particular, there is no dependence on projection dimension, whereas the extrinsic binning approach requires computing a *d*-dimensional grid comprising a number of cells in the order *O*(*r*^*d*^), that is, exponential dependence on dimension (Figure 1B). Thus, in cases where we expect to need a moderate number of dimensions (>10) to represent our data, intrinsic binning should be preferable to using extrinsic binning. Note that in standard neuroimaging literature where PCA is used for dimension reduction, standard choices for the embedding dimension range from 5–10 (Shine et al., 2019a) to 50 (Vidaurre et al., 2017).

Finally, we remark that the procedure of computing a transformed metric *D*′, for example, via the reciprocal *k*NN construction, and subsequently an overlapping cover of *X* via intrinsic binning, has the same inputs and outputs as the filtering and extrinsic binning approach of the standard Mapper pipeline. Therefore, these aspects of our overall pipeline comprise a self-contained module that can be incorporated into existing Mapper packages such as KeplerMapper or Giotto-Mapper, thus enriching their diverse functionality with scalability improvements toward qualitative and quantitative exploration of consortium-sized datasets.

#### Partial clustering and graph generation.

For partial clustering, we use the method described in the original Mapper literature (Singh et al., 2007). For each bin *B*_*i*_, we apply single linkage clustering using the native, high-dimensional metric *D*. Then we investigate the histogram of linkage values and set a cutoff threshold to be the first histogram bin edge at which the histogram bin count becomes zero. This threshold is then used to partition *B*_*i*_ into clusters.

Intuitively, if *B*_*i*_ contains two well-separated clusters, then this cutoff value would separate the clusters. As noted in Singh et al. (2007), this method has its limitations, namely, that if a bin contains clusters of differing densities, it tends to recover only the high-density cluster. However, this simple histogram-based method has worked sufficiently well for our purposes.

The output of the partial clustering step is an overlapping collection of bins *C*_0_, *C*_1_, …, *C*_*N*_. The final NeuMapper shape graph is generated by taking the *C*_*i*_ as nodes and inserting edges (*C*_*i*_, *C*_*j*_) whenever *C*_*i*_ and *C*_*j*_ share one or more data points.

#### Annotations.

Labels on data points are conveniently aggregated into annotations for each node of the shape graph. Specifically, given a shape graph on *N* nodes and *T* categorical labels, we construct an *N* × *T* annotation matrix where entry (*v*, *t*) counts the number of data points labeled *t* that belong to node *v*. The row vector (*v*, ·) comprises an annotation for node *v*. These annotations can be displayed as pie charts (Geniesse et al., 2019; Saggar et al., 2018) or used downstream in further analysis (see Results). While the labels themselves are typically supplied by the experiment design, we show below how these labels may be derived from meta-analysis to derive further insights into the data.

#### Parameter optimization.

As is standard in machine learning pipelines, parameter optimization for NeuMapper may be carried out via cross-validation. However, it is always appealing to obtain heuristics for good parameter initializations, and here we outline one such heuristic for optimizing parameters (*r*, *k*, *g*) that uses the autocorrelation structure of fMRI data. Specifically, given a data matrix *X*, we plot the autocorrelation function for each column of *X* and visually determine the “elbow,” that is, the number of lags at which the autocorrelation function becomes level. We multiply this number by the sampling period of the dataset to obtain a *critical lag* τ in units of seconds. In the fMRI context, autocorrelation is naturally present due to the hemodynamic response function (HRF), and it is desirable to view data at a scale which incorporates signal that is not just driven by HRF. Toward this goal, we specify a percentage value *α* and set the criteria—denoted AutoCorrCrit—that an output shape graph should have at least *α*% nodes containing data points that were acquired at least τ seconds apart, that is, are less susceptible to the HRF. We set *τ* = 11*s* (corresponding to the HRF peak; Lindquist et al., 2009) and *α* = 25% for both datasets.

The procedure outlined above heuristically attempts to mitigate the dependency of NeuMapper shape graph nodes on autocorrelated samples. However, thus far we do not have any conditions guaranteeing that the output shape graphs will be sufficiently connected for carrying out downstream analysis by using network science tools. To this end, we introduce an additional percentage value *β* and require that for group-level analysis, each shape graph contains at least *β*% of its nodes in its largest connected component. We set *β* = 50% for both datasets. To ensure consistency in group-level analysis, we require a consensus (*r*, *k*, *g*) triplet that can be used to generate shape graphs for each dataset, that is, for data acquired under the same scanning parameters. In summary, we first obtain parameters for each shape graph in the dataset according to *α*, obtain a consensus (*r*, *k*, *g*) triplet for the full dataset, and finally perturb the consensus triple (if necessary) to satisfy the connectivity constraint *β*. While other strategies may also be invoked to satisfy our criteria, we found that this order of operations worked sufficiently well.

In detail, the optimization at the level of a single subject is carried out as follows. We first specify a broad range of values for the *r* parameter, and choices of small initial *k* and *g* parameters. For each value of *r*, we carry out the following procedure:

- Compute a shape graph with the initial values for *k* and *g*.
- Verify that AutoCorrCrit is satisfied. If not, increment *k* ← *k* + 1 and *g* ← *g* + 3.
- Iterate until AutoCorrCrit is satisfied.

More specifically, for the *r* parameter, we explored 10 different values evenly spaced along the interval [floor(0.1 · *n*), floor(0.3 · *n*)], where *n* is the number of time points in the dataset. For the *k* and *g* parameters, we used small initial values of *k* = 3 and *g* = 25, respectively. The step sizes for incrementing *k*, *g* were chosen to be the smallest integers such that perturbing the corresponding parameters produced observable changes to the shape graphs.

Multiple *r* values may have the same optimal (*k*, *g*) parameters. To reduce these choices down to a manageable number, we cluster the different optimal *k* values (equivalently *g* values, as we increment *k*, *g* together) using the classical DBSCAN density-based clustering algorithm. We then discard all but the top three largest clusters of optimal *k* values. Typically, each cluster will have a unique *k* value, but to ensure this programmatically, we select the minimum *k* value for each cluster. Finally, for each cluster we record the most frequently occurring *r* value. This yields a total of three optimal (*r*, *k*, *g*) triplets for a data matrix *X*.

We repeat this procedure for the data matrix for each subject. To obtain consensus, we use a simple voting procedure to select three (*r*, *k*, *g*) triplets that occur most frequently among the optimal triples for each subject. This procedure returns three consensus (*r*, *k*, *g*) triplets at the group level. We observed that these three optimal parameter triples correspond to three different scales of shape graphs, and the triplet with intermediate values of (*r*, *k*, *g*) best corresponds to a mesoscale view with interesting structure.

Having chosen a consensus (*r*, *k*, *g*) triplet, we then verified for each dataset that each of the shape graphs had over *β*% of the nodes in the largest connected component. If not, we increased *k* and decreased *r* (both steps incorporate more global information) in small steps of 3 and 5, respectively, until all graphs had a sufficient fraction of nodes in the largest connected component. Note that we could not perform this step before choosing the consensus triplet, as the procedure for obtaining consensus could result in some graphs no longer satisfying the connectivity criterion. For Dataset 1, the consensus triplet yielded *r* = 192, *k* = 8, *g* = 40. For these parameters, each of the shape graphs satisfied the connectivity criterion, and no further parameter tuning was needed. and we directly used these parameters for generating graphs via NeuMapper. For Dataset 2, the mesoscale triple yielded *r* = 260, *k* = 6, *g* = 34. However, visual inspection of the graphs generated using these parameters revealed highly disconnected graphs for multiple participants, suggesting that the data for these participants had different spatial characteristics from that of the rest of the group. After carrying out parameter tuning as described above, the final (*r*, *k*, *g*) triple that we used for Dataset 2 was (240, 18, 34).

### Methods for Analyzing Shape Graph Annotations

Toward qualitative and interactive exploration, we created visualizations of the shape graphs obtained by NeuMapper using the DyNeuSR platform (Geniesse et al., 2019) and used its node annotation features to demonstrate insights into behavior (Figure 2). On the quantitative side, we show that shape graphs are compressed graph representations of the data on which post hoc analysis via network science tools can be carried out efficiently.

#### Community detection tools.

We used two standard community detection methods in our analysis: modularity (Newman, 2006), which tracks the appearance of densely connected groups of vertices with sparse connections across groups, and core-periphery (Borgatti & Everett, 2000), which tracks the appearance of a centrally located group of vertices with dense connections to the whole graph that is surrounded by a periphery with sparse connections. Modularity of each Mapper-generated graph was computed as follows. Using the ground-truth labels, each node was assigned to a community (one of the four tasks) via winner-take-all voting. The modularity of this community assignment was then computed using the standard *Q*-score (Newman, 2006). For core-periphery, we used the score derived in Everett and Borgatti (2000), Newman (2006), and Rubinov et al. (2015) and validated in Saggar et al. (2018). Specifically we used the implementation core_periphery_dir provided in the Brain Connectivity Toolbox (Rubinov & Sporns, 2010). In this implementation, each node is given a binary core-periphery assignment. The coreness of each task was defined to be the fraction of time points for each task that belonged to core nodes.

#### Optimal transport.

The main tool from OT that we use is the *1-Wasserstein distance*, also known as the Earth Mover’s Distance (Rubner et al., 2000). The origin of OT is attributed to the work of Monge in the late 1700s and that of Kantorovich in the 1940s.

The 1-Wasserstein distance is defined in our setting as follows. We start with a connected shape graph *G* = (*X*, *E*) (restricting to the largest connected component as necessary), matrix of graph geodesic distances *D*′, and probability distributions *p*, *q* defined on the vertices of *G*. For shape graphs, probability distributions arise naturally from task or NeuroSynth-derived annotations: for a given annotation *S* and any shape graph vertex *v*, we first compute the proportion of data points in *v* that are annotated by *S*, and then normalize these proportions over all of *G* to get a vector of length *n* × 1 (where *n* = |*X*|) with nonnegative numbers that sum to 1. Following this procedure for two separate annotations yields two annotation-derived probability distributions *p* and *q*. Intuitively *p*, *q* correspond to distributions of “mass” on the points of *G*. Next we consider the set of all joint probability distributions with marginals *p*, *q*, denoted Π(*p*, *q*). Each *μ* ∈ Π(*p*, *q*) is an *n* × *n* matrix with nonnegative entries summing to 1 and row and column sums equal to *p* and *q*, respectively. Finally, the 1-Wasserstein distance is defined as:

$$d_{OT}\left(p,q\right)≔\min_{\mu \in \Pi \left(p,q\right)}\sum_{i,j=1}^{n}D_{ij}\mu_{ij}.$$

This is a linear program that can be solved using standard techniques in linear programming. We use the Matlab wrapper written by Antoine Rolet for the C++ implementation provided in Bonneel et al. (2011).

This OT distance is a bona fide metric between probability distributions that calculates the cost of transforming one distribution into another while taking into account the ground distances between the points supporting the two distributions. In the language of network flows (Ahuja et al., 1989), computing this metric amounts to solving a minimum cost flow from one distribution to another. For the NeuMapper setting, *d*_*OT*_ is a principled method for computing second-order information from different annotations. Figure 3 shows the use of pairwise *d*_*OT*_ followed by an MDS projection to get a 2-D summary of the separation between task annotations on shape graphs across our two datasets.

#### Generating annotations via meta-analysis.

To better anchor the NeuMapper-generated graphs into neurobiology, we generated a second set of node-level annotations using topic association maps based on the NeuroSynth decoding framework (Yarkoni et al., 2011). Topic association maps were downloaded from neurosynth.org using the NeuroSynth *v4-topics-50* database—a set of 50 topics extracted from all abstracts in the NeuroSynth database as of July 2015. For the purpose of this study, we limited our analysis to the topic association maps (i.e., Topics 010, 022, 011, 042) most relevant to the four tasks used in the CMP experiment (i.e., resting state, working memory, video, math), and a fifth topic association map corresponding to “task-positive” cognition (i.e., Topic 002).

For each topic association map, we extracted the mean values from 333 predefined cortical ROIs based on the Gordon et al. (2016) atlas, and transformed the resulting 3-D NIFTI image into a 2-D topic association matrix. These two steps were performed simultaneously, using the “NiftiLabelsMasker” object from the Nilearn software package (Abraham et al., 2014). Note, the atlas used to prepare the fMRI data for input into NeuMapper was processed using the Shine 375 atlas (as described in Data Acquisition section), which includes the same 333 cortical regions from the Gordon et al. (2016) atlas used here to process the topic association maps. Thus, before computing any correlations between the fMRI data and the NeuroSynth topic association maps, we extracted data corresponding to the same 333 cortical regions from each of the preprocessed fMRI data matrices. Further, the resulting data matrices were converted to *z*-scores (computed for each subject, separately), and individual ROIs with zero variance were excluded from further analysis.

Next, for each subject, we computed “framewise” correlations between each topic association map and every time frame in the data matrix. This yielded a frame-by-topic correlation matrix that was then used to aggregate node-level annotations. For each node, the topic correlation scores corresponding to time frames associated with the node were extracted from the precomputed frame-by-topic correlation matrix, and then further restricted to include only those within the top 99th-percentile of strongest positive correlations associated with the node. Finally, to generate the node-level annotations, we counted the number of retained positive frame-by-topic correlations associated with each node, and then used these node-by-topic association counts as the annotations for each node. This yielded a final node-by-topic annotation matrix that could then be used to color the NeuMapper-generated graphs and perform further analysis (as described in Anchoring Shape Graphs Into Known Cognitive Constructs section).

#### Overreflection and synchronization measures.

To better understand how the “decoded” cognitive topic annotations relate to “expected” task structure and experimental task performance, we developed two new measures to quantify the amount of overlap between NeuroSynth topic annotations and experimental task labels.

Overreflection/mismatch was computed as follows. For each subject, the experimental task labels (R, M, V, A) were used to compute a node-by-task annotation matrix $\hat{\Theta }$, from which we obtained a condensed two-column node-by-task annotation matrix Θ by summing the M, V, and A task columns. We also used NeuroSynth task-positive and task-negative topic annotations (Topic 002 and Topic 010) to compute a two-column node-by-topic association matrix Φ. We then computed the matrix product Φ^*T*^Θ and normalized this 2 × 2 matrix by its sum, formally written as Φ*^T^*Θ/〈Φ^*T*^Θ, 1〉_*F*_ where 〈·〉_*F*_ denotes the matrix Frobenius product. Each entry of this matrix corresponds to the overlap between an empirical (NeuroSynth-decoded) annotation and an expected (experimental task-derived) annotation. The overreflection score was then defined to be the off-diagonal entry corresponding to the NeuroSynth-decoded task-positive annotation and the experimental rest annotation.

Synchronization/match between the empirical and expected rest annotations and the empirical/expected task annotations was computed in two stages: once for aggregated tasks, and once for the individual memory task. First, synchronization at the task-aggregated level was taken to be the trace (i.e., the sum of the diagonal) of Φ^*T*^Θ/〈Φ^*T*^Θ, 1〉_*F*_. Second, for synchronization of the individual memory task, we used NeuroSynth rest and working memory topic annotations (Topic 010 and Topic 022) to compute a two-column node-by-topic annotation matrix $\bar{\Phi }$. We also extracted the R, M columns from $\hat{\Theta }$ to obtain a corresponding two-column node-by-task annotation matrix $\bar{\Theta }$. We then computed a synchronization score by taking the trace of the normalized matrix product $\bar{\Phi }$^*T*^$\bar{\Theta }$/〈$\bar{\Phi }$^*T*^$\bar{\Theta }$, 1〉 as before.

### Validation and Replicability Analysis

#### Phase-randomized surrogates.

To ground NeuMapper in a randomization framework, we validated our pipeline against null datasets produced via Fourier phase randomization (Theiler et al., 1992). Phase randomization takes a collection of time series data, applies a discrete Fourier transform to each time course, adds a uniformly distributed random phase to each frequency, and then inverts the discrete Fourier transform. Phase randomization produces stationary, linear, Gaussian null data that—by the Wiener-Khintchine theorem—preserves the autocorrelation structure of the original data (Khintchine, 1934; Prichard & Theiler, 1994; Wiener, 1930). Thus by testing against phase-randomized surrogates, we test against the possibility that our results are driven by stationarity, linearity, Gaussianity, or any combination of these properties (Liégeois et al., 2017). We validated our framework on phase-randomized surrogates from both Datasets 1 and 2 (Supporting Information Figure S3), using 10 random surrogates for the data coming from each participant. Surrogate data were generated using the code accompanying (Liégeois et al., 2017).

#### Parameter perturbation.

To ensure that our results are stable to parameter perturbation, we repeated several of our analysis pipelines on a grid of (*r*, *k*, *g*) parameters surrounding the chosen parameters for Datasets 1 and 2. Results for the correlations between modularity and task performance as well as the core-periphery structure are presented in Supporting Information Figure S4. Overall, we find that our results are stable to nearby parameter perturbations, with the gain parameter showing the most discrete changes. Future work should clarify the role of the gain parameter; some of our tests indicated that at least for methods based on *k*NN constructions, the gain parameter could be removed altogether, leaving only the (*r*, *k*) parameter pair to be tuned.

## ACKNOWLEDGMENTS

The authors thank the reviewers for their helpful comments. We also thank the research staff and students for their support in data collection, especially, Javier Gonzalez-Castillo for Dataset 1 and Amber Howell, Sahar Jahanikia, Rafi Ayub, and Hua Xie for Dataset 2.

## SUPPORTING INFORMATION

Supporting information for this article is available at https://doi.org/10.1162/netn_a_00229. Dataset 1 was originally collected by Gonzalez-Castillo et al. (2015) and is available for download from the XNAT Central public repository (https://central.xnat.org; Project ID: FCStateClassif). The preprocessed data used for Dataset 2 is available upon reasonable request.

Custom Matlab scripts used to generate and analyze the shape graphs can be found at https://github.com/braindynamicslab/neumapper. The DyNeuSR Python package used to visualize the shape graphs can be found at https://github.com/braindynamicslab/dyneusr.

## AUTHOR CONTRIBUTIONS

Caleb Geniesse: Conceptualization; Investigation; Methodology; Software; Validation; Visualization; Writing – original draft; Writing – review & editing. Samir Chowdhury: Conceptualization; Investigation; Methodology; Software; Validation; Visualization; Writing – original draft; Writing – review & editing. Manish Saggar: Conceptualization; Data curation; Formal analysis; Funding acquisition; Investigation; Methodology; Project administration; Resources; Software; Supervision; Validation; Visualization; Writing – review & editing.

## FUNDING INFORMATION

Manish Saggar, National Institute of Mental Health (https://dx.doi.org/10.13039/100000025), Award ID: MH-119735. Manish Saggar, National Institute of Mental Health (https://dx.doi.org/10.13039/100000025), Award ID: MH-104605. Manish Saggar, Stanford University MCHRI Faculty Scholar.

## TECHNICAL TERMS

Mapper: TDA-based technique for generating graph representations that identify meaningful subgroups of high-dimensional data.
Topological data analysis (TDA): Applied mathematical approaches for analyzing datasets—using techniques from algebraic topology—that learn and leverage information about the shape of complex data.
Shape graph: Compressed network representation generated by Mapper; nodes correspond to local clusters and edges connect clusters that share data points.
Dimensionality reduction: Process of selecting and extracting features from data to reduce the number of variables under consideration.
Partial clustering: Applying a clustering algorithm to a subset of data points. Used to obtain cluster bins from cover bins.
Optimal transport (OT) metric: A measure of similarity (or dissimilarity) between two probability distributions (e.g., 1-Wasserstein distance). When comparing distributions on a graph, these metrics consider both global and local properties.
NeuroSynth: A meta-analytic database of probabilistic mappings between cognitive (e.g., terms, topics) and neural states (i.e., activations) that can be used for a broad range of neuroimaging applications.
Mesoscale structure: A description of network structure in terms of node density and within-group connectivity, for example, the clustering of nodes into different groups (i.e., modularity) or the partitioning of nodes into dense core and sparse peripheral regions (i.e., core-periphery).
Binning: A step of Mapper-type algorithms that partitions a dataset. Neighboring partition blocks are encouraged to have overlaps.
Cover bins: A collection of overlapping sets returned by the binning procedure.
Cluster bins: A refined collection of overlapping sets produced by clustering each set in the collection of cover bins independently.
Geodesic distance matrix: A distance matrix describing the pairwise shortest path lengths between nodes in a graph.
*k*-nearest neighbor (*k*NN) graph: A graph built on point cloud data where nodes correspond to data points, and edges connect a node to its *k* (a positive integer) closest data points.
Reciprocal *k*NN: A *k*-nearest neighbors graph created by dropping nonsymmetric neighbors (i.e., two points are only considered neighbors if each is a *k*-closest neighbor of the other). Found to better respect local density when connecting neighbors than standard *k*NN.
Intrinsic binning: A binning method that uses landmarks and distances from these landmarks to partition data points.
Farthest point sampling (FPS): An algorithm for selecting a well-separated set of landmark points from a dataset.
Distance matrix: A square matrix describing the pairwise distances (e.g., Euclidean) between points in a dataset.
Extrinsic binning: A binning method that uses a grid to partition data points. Good for cases where the ambient space is low dimensional.
