# Supplementary material for: NeuMapper: A scalable computational framework for multiscale exploration of the brain’s dynamical organization
Source: Netw Neurosci. 2022 Jun 1;6(2):467–98. doi: 10.1162/netn_a_00229 (PMC9207992; doi:10.1162/netn_a_00229)
Supplement: Supplementary file 1 [file netn-06-467-s001.pdf]

Supplementary Information for

NeuMapper: A Scalable Computational Framework for Multiscale Exploration of the Brain's Dynamical Organization

Caleb Geniesse[1,2*], Samir Chowdhury[2*], and Manish Saggar[2†]

[1]Biophysics Program, Stanford University, Stanford, CA, USA

[2]Department of Psychiatry and Behavioral Sciences, Stanford University, Stanford, CA, USA

* Equal contribution

† Corresponding Author: saggar@stanford.edu

Keywords: TDA, Mapper, Optimal transport, Multitask fMRI, Ongoing cognition, NeuroSynth

Supplementary Figures

Here we provide the following additional materials to clarify details in the main text:

- Supplementary Fig. 1 | Adaptations to the standard Mapper approach for avoiding low dimensional projection.
- Supplementary Fig. 2 | Mesoscale structure of shape graphs from individual datasets.
- Supplementary Fig. 3 | Phase-Randomized null surrogates from Datasets 1 and 2 lose the structure seen in the true data.
- Supplementary Fig. 4 | Modularity-behavior correlations remain stable to parameter perturbation.
- Supplementary Fig. 5 | Shape graphs can reveal temporal transitions at the level of individual time frames.
- Supplementary Fig. 6 | Comparison of kNN, reciprocal kNN, and NeuMapper.
- Supplementary Fig. 7 | Scalability of NeuMapper.
- Supplementary Fig. 8 | Examining the effect of dimension reduction on data from Dataset 1, S_{01}.

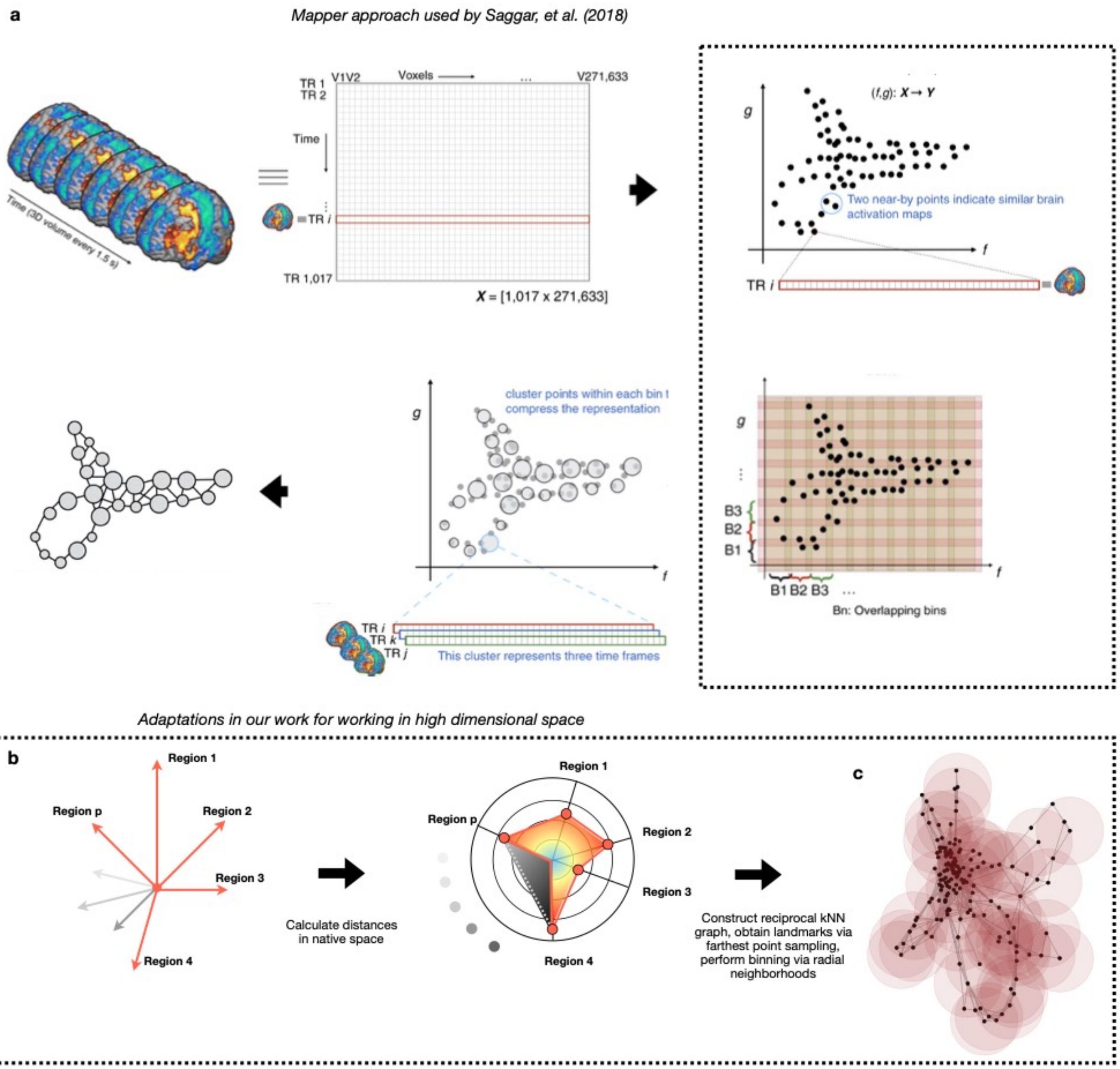

Supplementary Fig. 1 | Adaptations to the standard Mapper approach for avoiding low-dimensional projection. [a] The standard Mapper approach requires an initial embedding of the high-dimensional input data into a low-dimensional space. This embedding space is then partitioned into a set of overlapping bins (i.e., hypercubes) such that each data point is assigned to one or more bins based on their position in the low-dimensional embedding space. To recover some of the information lost during the projection into the low-dimensional space, points in the

original high-dimensional space are grouped together if they belong to the same bin in the embedding space, and clustering is applied to each group to further refine each bin. This step is referred to as partial clustering. Finally, a shape graph is constructed by taking the nodes to be the refined bins, and adding edges between nodes if they share one or more of the same data point(s). Changes introduced by the NeuMapper approach are shown in the dashed box. Note that the inputs and outputs to the dashed boxes are otherwise consistent. **[b]** Rather than first projecting the input data into a low-dimensional space, NeuMapper instead groups nearby points together directly in the high-dimensional space, possibly by using a nonlinear transformation of the distances between the data points. In our demonstrated applications, we utilize pairwise geodesic distances on a reciprocal kNN graph that approximate the nonlinear geometry of the landscape of brain configurations across time. Dimensions correspond to parcellated brain volumes. **[c]** To perform grouping in high-dimensional space without constructing exponentially many hypercubes, NeuMapper employs a data-driven *intrinsic* binning strategy that first constructs landmarks on the data, and then defines the bin centered at each landmark to comprise the subset of data points within a certain distance from the landmark. In our applications of NeuMapper, we perform this intrinsic binning on the reciprocal kNN graph with graph geodesic distances.

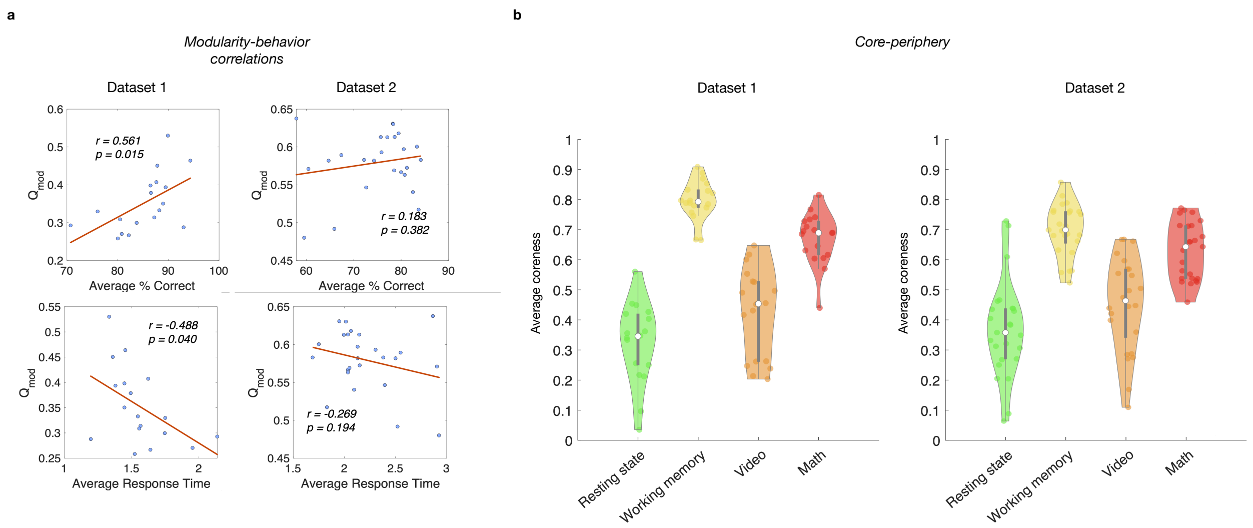

Supplementary Fig. 2 | Mesoscale structure of shape graphs from individual datasets. [a] Modularity-behavior correlations obtained for each dataset, separately. For Dataset 1 (n = 18) we observed that the task-based modularity of shape graphs is significantly positively correlated with average task accuracy ($r = 0.561, p = 0.015$) and significantly negatively correlated with response time ($r = -0.488, p = 0.040$). For Dataset 2 (n = 25) we observed that the task-based modularity was positively correlated with average task accuracy ($r = 0.183, p = 0.382$) and negatively correlated with response time ($r = -0.269, p = 0.194$). Although these correlations were not significant, the trend lines follow the expected positive and negative trends, respectively. **[b]** Core-periphery structure observed in each dataset, separately. One-way ANOVA revealed significant effects of task in Dataset 1 ($F(3,68) = 67.0, p = 2.9 \cdot 10^{-20}$) and Dataset 2 ($F(3,96) = 35.6, p = 1.5 \cdot 10^{-15}$). For both datasets, tasks with high cognitive load such as working memory or math were associated with nodes found relatively deep inside the core of the shape graphs, whereas resting state nodes were relatively more peripheral.

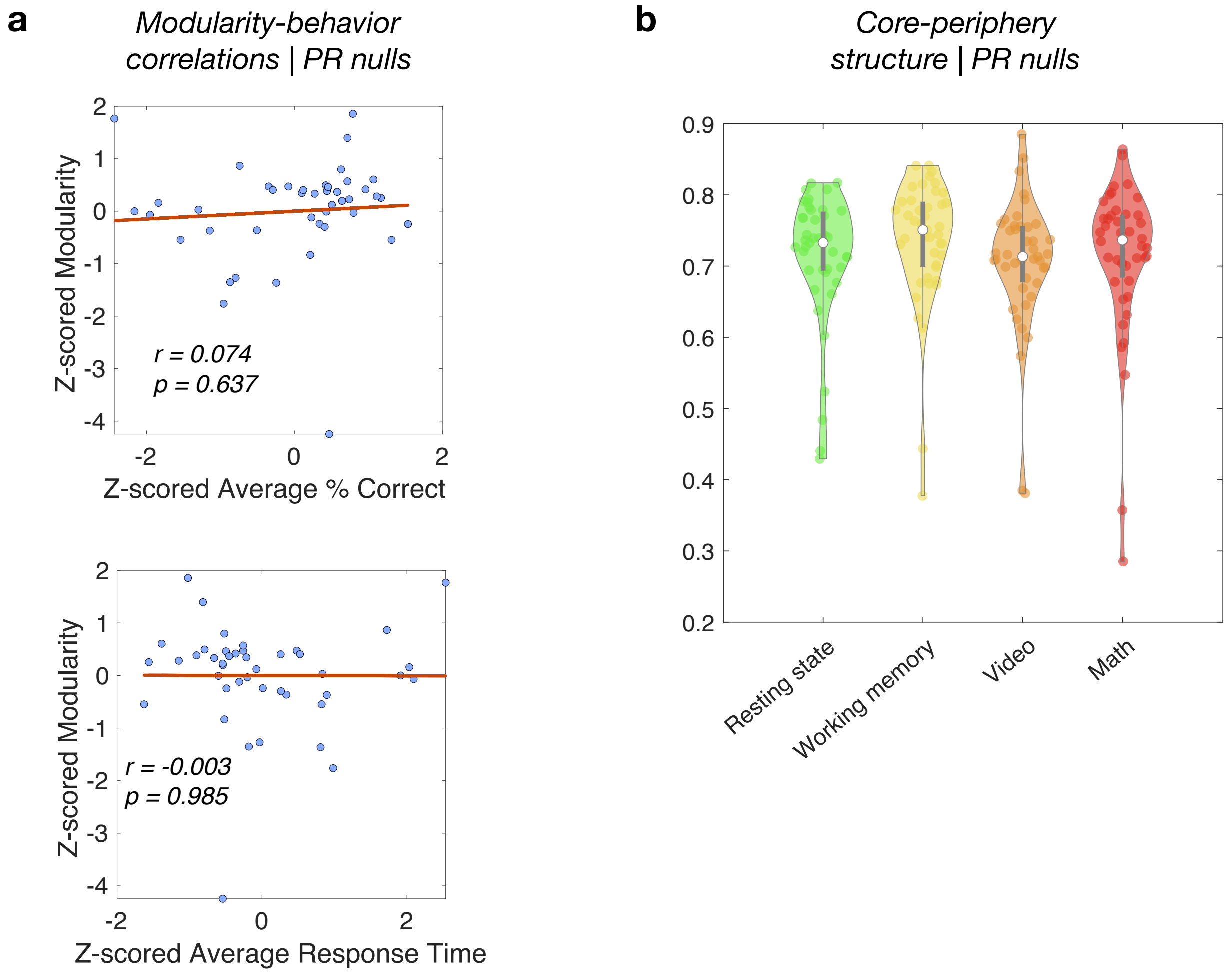

Supplementary Fig. 3 | Phase-Randomized null surrogates from Datasets 1 and 2 lose the structure seen in the true data. Here we see that **[a]** modularity-behavior correlations become insignificant and **[b]** differences in core periphery structure are lost ($F(3,168)=0.74$, $p = 0.53$).

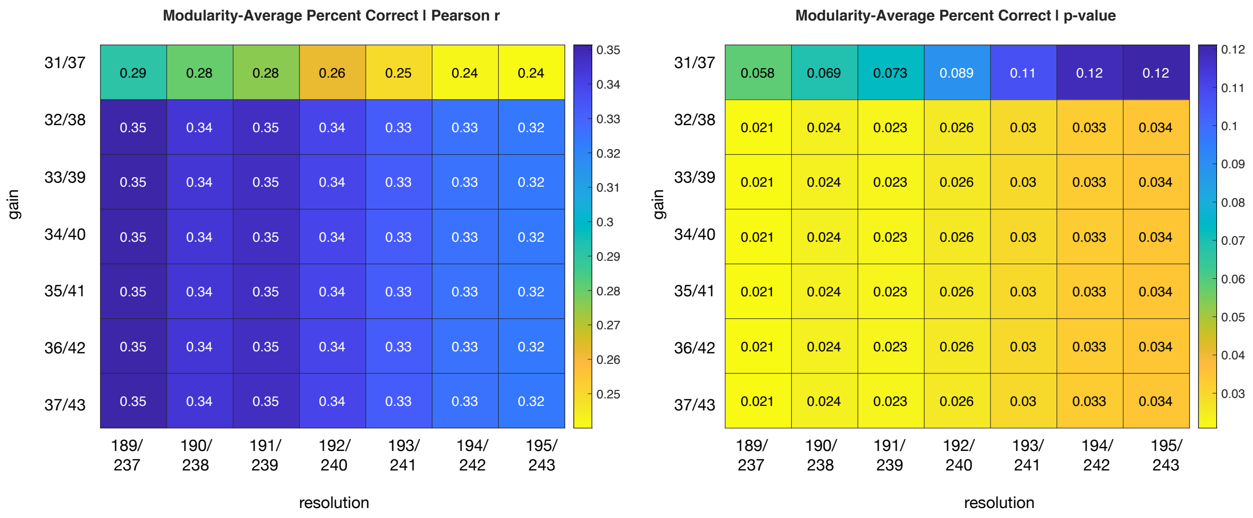

Supplementary Fig. 4 | Modularity-behavior correlations remain stable to parameter perturbation. The axis labels (e.g., 31/37) correspond to the parameter values used for Dataset 1 and 2, respectively. The results were z-scored and combined to produce this table. Note that in reported results, we used resolutions 192, 240 and gains 34, 40 for Datasets 1 and 2, respectively.

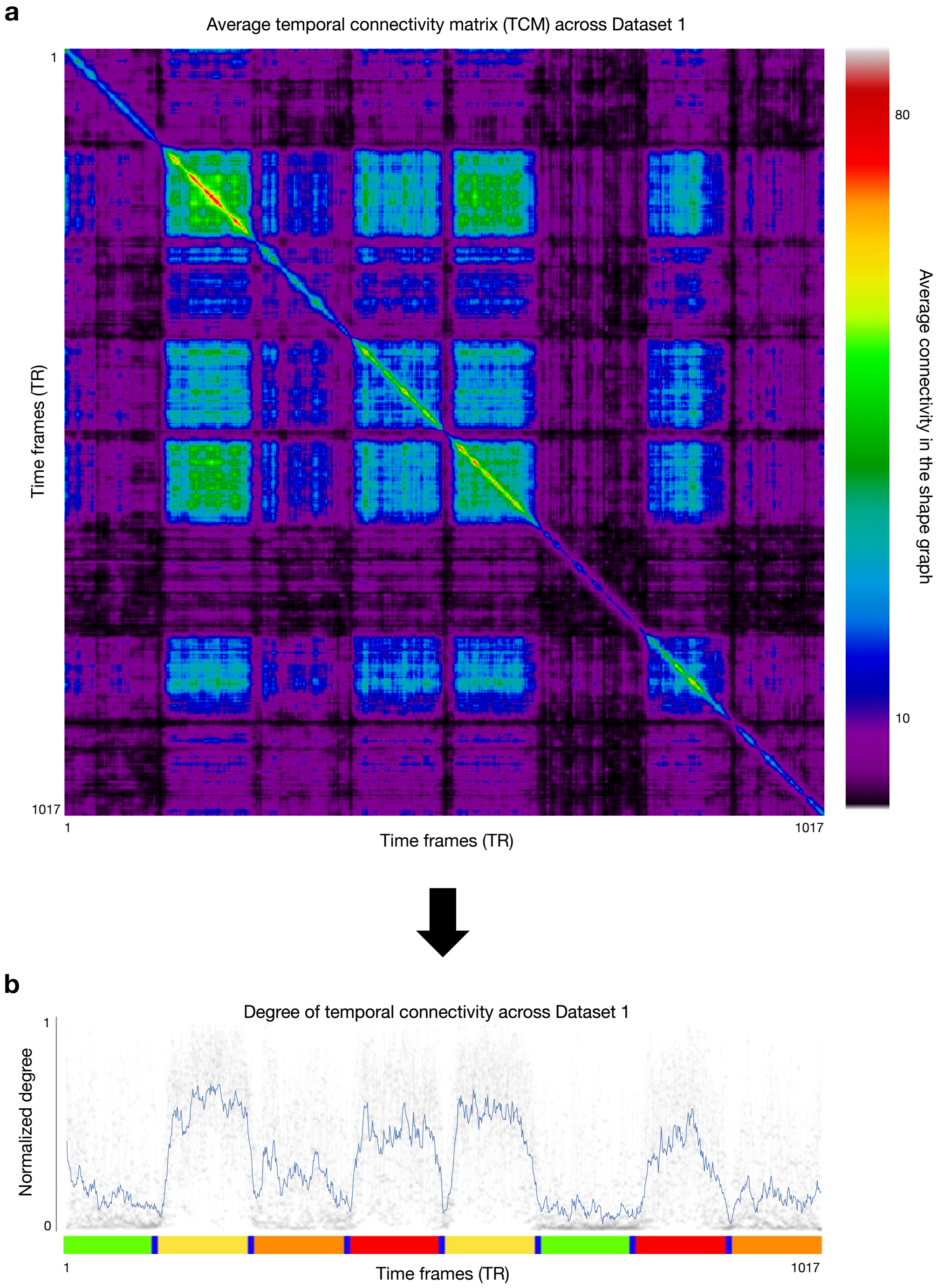

Supplementary Fig. 5 | Shape graphs can reveal temporal transitions at the level of individual time frames. The temporal connectivity matrix (TCM) shows how individual time frames are connected in the shape graph, and the degree of nodes in the TCM can reveal task-

evoked transitions in brain activity at the highest temporal resolution (i.e., individual time frames). **[a]** Here we show the average TCM across all 18 participants from Dataset 1. **[b]** The average degree of nodes in the TCM across all 18 participants (blue solid line) recovers the task-block structure of the continuous multitask paradigm. Here we also show the degree of nodes in the TCM for individual participants (gray dashed line). As expected, the temporal evolution of the group-averaged degree of connectivity visually recovers the task-block structure (i.e., higher degrees evoked by and maintained during non-rest task blocks) and between-task transitions (i.e., lower degrees spanning the between-task instructional periods) of the CMP at the highest temporal resolution (i.e., changes in degree occurring within a few time frames of the onset and offset of tasks).

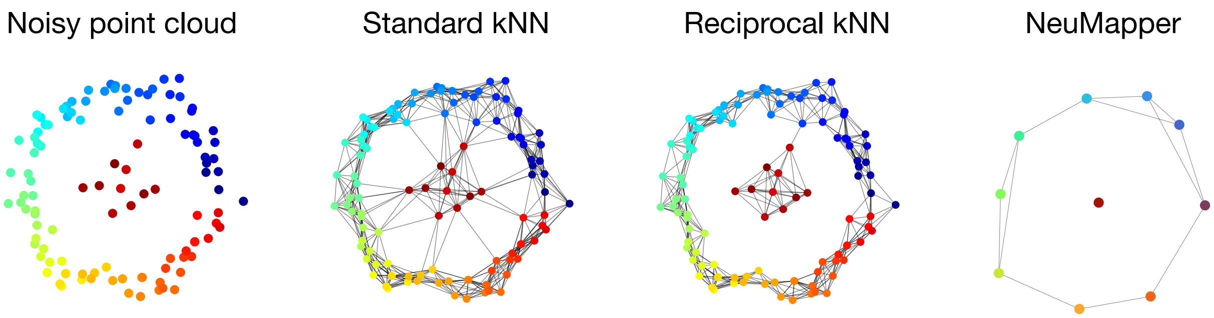

Supplementary Fig. 6 | Comparison of kNN, reciprocal kNN, and NeuMapper. Here, we show visually how using the reciprocal kNN improves our results compared to the standard (nonreciprocal) kNN. In this example, the noisy point cloud includes an outer ring of points and a central island of a much smaller subset of (n = 10) points. Each graph was constructed using the same noisy point cloud data and the same parameter (k = 12). Notably, the standard kNN fails to separate the outer ring and the central cluster, forming many connections between the two regions of space, since only ten points reside in the central cluster yet the standard kNN requires each point be connected to at least k = 12 neighbors. In contrast, the reciprocal kNN removes most of the incorrect connections that are not reciprocated by points in the denser outer ring, allowing points in the less dense central cluster to form less than k = 12 neighbor connections. Finally, NeuMapper produces a compressed version of the reciprocal kNN graph via binning and eliminates the final incorrect connection by partial clustering based on the original Euclidean space.

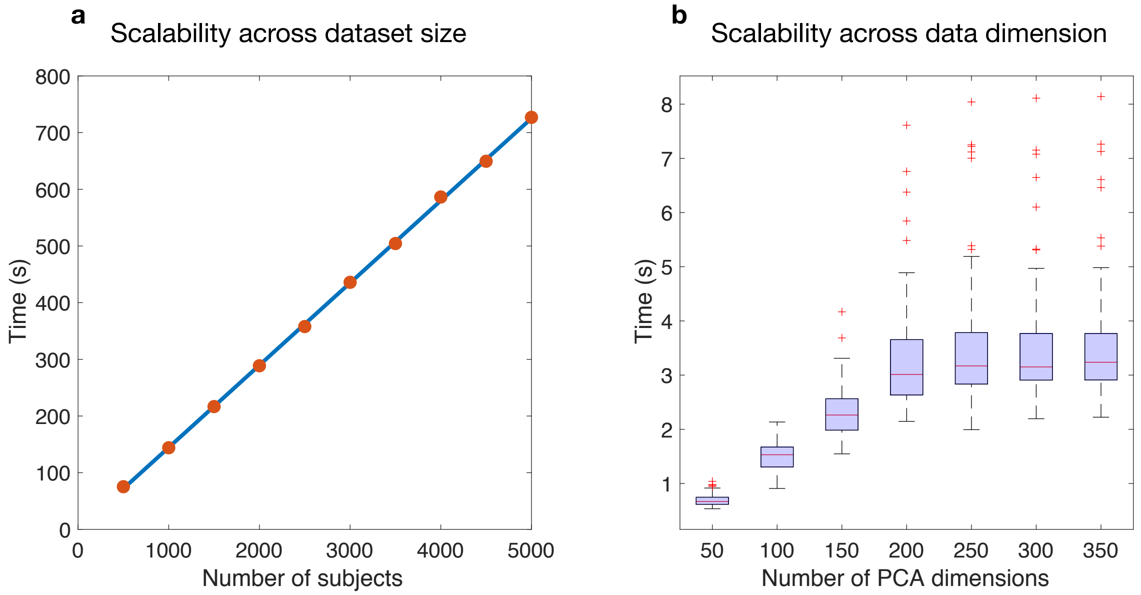

Supplementary Fig. 7 | Scalability of NeuMapper. [a] Toward demonstrating applicability of NeuMapper on consortium-sized datasets, we generate synthetic data from Dataset 1 by phase-randomization. Each synthetic data matrix has the same dimensions as one of the subjects from Dataset 1 (i.e., 1017 x 375), and we generate 10 datasets containing 500, 1000, 1500, …, 5000 of these data matrices. We then compute NeuMapper graphs for each dataset using Matlab's parfor with eight workers. For 5000 data matrices, the computation takes just above 10 minutes. **[b]** NeuMapper shows excellent scalability with respect to data dimension. Here we generated 100 phase-randomized surrogate data matrices of shape 1017 x 375, applied PCA projection with 50, 100, …, 350 PCA components, computed NeuMapper graphs, and saved the runtime of each NeuMapper computation. The runtimes at each dimension are displayed in the boxplots.

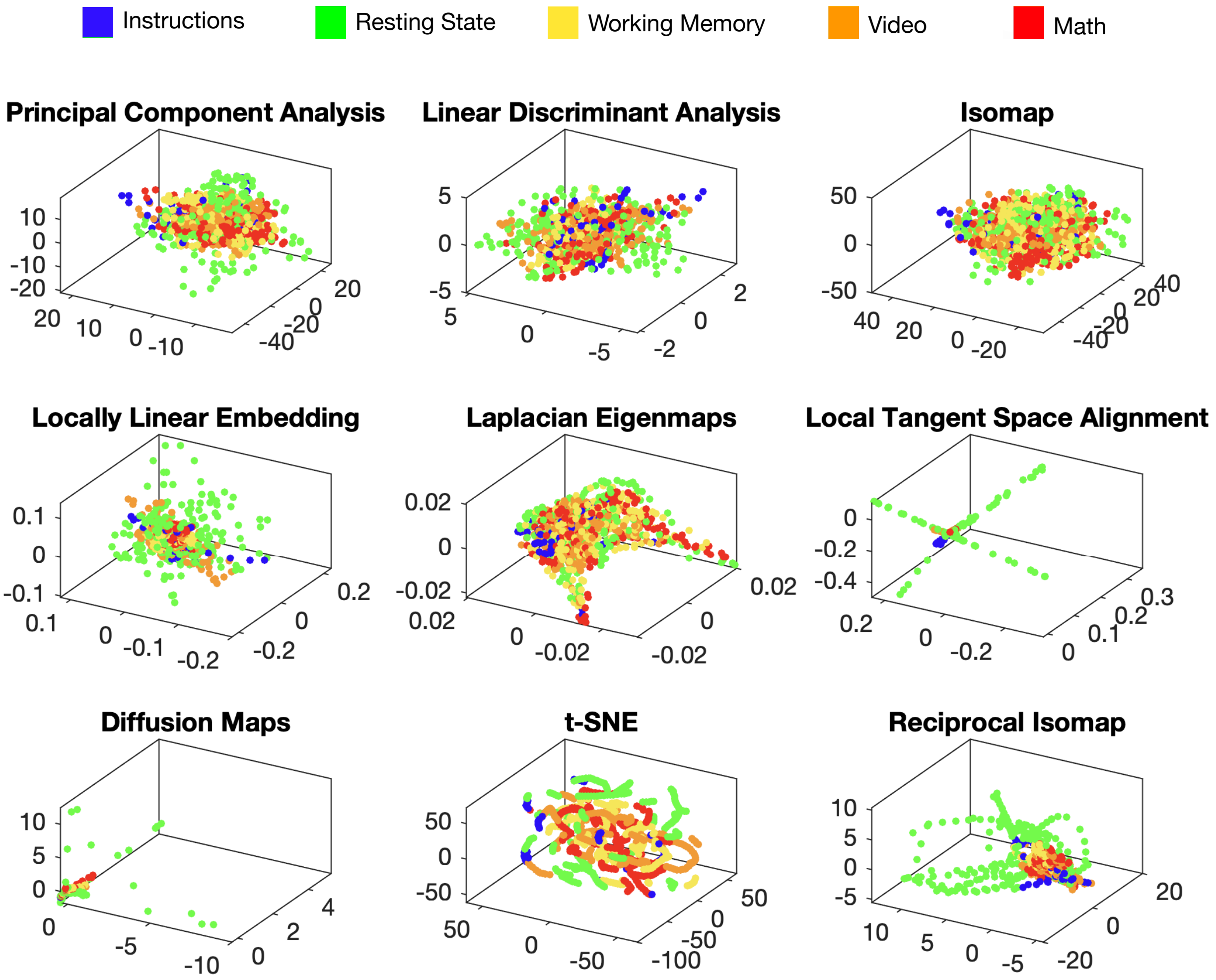

Supplementary Fig. 8 | Examining the effect of dimension reduction on data from one participant S_{01} of Dataset 1. To obtain a coarse understanding of the geometry of our data, we took a single data matrix from an exemplar subject and inspected the results from applying linear and nonlinear dimension reduction techniques to obtain 3D point clouds. We also took one of the simplest nonlinear methods that relies on a kNN graph construction— the Isomap algorithm (Tenenbaum et al., 2000)—and repeated the algorithm after replacing the kNN graph with a reciprocal kNN graph construction. The result is illustrated in the last panel titled Reciprocal Isomap. A visual inspection of Isomap and Reciprocal Isomap suggests that to improve task

separability, one should use a method based on a reciprocal kNN construction. Among the other techniques, Locally Linear Embedding and Local Tangent Space Alignment both show some interesting structure. However, a closer look into the 3D plots suggests that these embeddings show worse task separability than Reciprocal Isomap.
